# Functionalized Cellulose Nanocrystals as Active Reinforcements for Light-Actuated 3D-Printed Structures

**DOI:** 10.1021/acsnano.2c05628

**Published:** 2022-10-18

**Authors:** Luca A. E. Müller, Anita Zingg, Andrea Arcifa, Tanja Zimmermann, Gustav Nyström, Ingo Burgert, Gilberto Siqueira

**Affiliations:** † Cellulose and Wood Materials Laboratory, Empa, Swiss Federal Laboratories for Materials Science and Technology, 8600 Dübendorf, Switzerland; ‡ Wood Materials Science, Institute for Building Materials, ETH-Zürich, 8093 Zürich, Switzerland; § Surface Science & Coating Technologies, Empa, Empa, Swiss Federal Laboratories for Materials Science and Technology, 8600 Dübendorf, Switzerland; ∥ Department of Health Sciences and Technology, ETH Zürich, 8092 Zürich, Switzerland

**Keywords:** cellulose nanocrystals, 3D printing, photoresponsive, azobenzene, mechanical adaptation

## Abstract

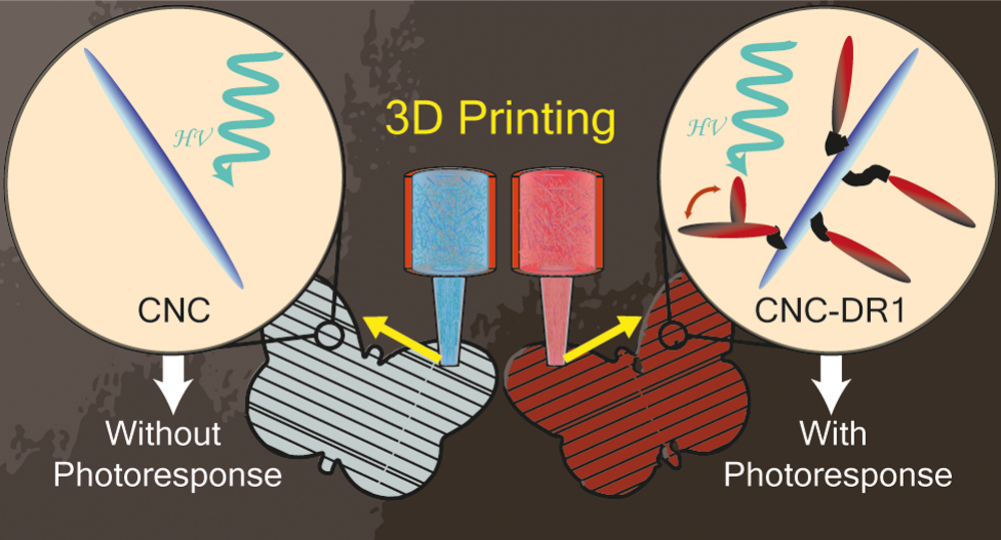

Conventional manufacturing techniques allow the production of photoresponsive cellulose nanocrystals (CNC)-based composites that can reversibly modify their optical, mechanical, or chemical properties upon light irradiation. However, such materials are often limited to 2D films or simple shapes and do not benefit from spatial tailoring of mechanical properties resulting from CNC alignment. Herein, we propose the direct ink writing (DIW) of 3D complex structures that combine CNC reinforcement effects with photoinduced responses. After grafting azobenzene photochromes onto the CNC surfaces, up to 15 wt % of modified nanoparticles can be introduced into a polyurethane acrylate matrix. The influence of CNC on rheological properties allows DIW of self-standing 3D structures presenting local shear-induced alignment of the active reinforcements. The printed composites, with longitudinal elastic modulus of 30 MPa, react to visible-light irradiation with 30–50% reversible softening and present a shape memory behavior. The phototunable energy absorption of 3D complex structures is demonstrated by harnessing both geometrical and photoresponsive effects, enabling dynamic mechanical responses to environmental stimuli. Functionalized CNC in 3D printable inks have the potential to allow the rapid prototyping of several devices with tailored mechanical properties, suitable for applications requiring dynamic responses to environmental changes.

## Introduction

Advancing sustainability in a technological world requires the development of lightweight devices that can reach peak mechanical performances while being composed of renewable and sustainable materials. As a result, in the last years, strong attention has been paid to biomimicry and bioinspiration since living organisms can rely on biological materials exhibiting highly optimized performances.^1^ Despite evolving with a limited palette of ingredients, such materials can often reach outstanding mechanical properties and demonstrate functional responses to external stimuli under demanding environmental conditions. A material like wood possesses highly anisotropic specific strength and stiffness,^2^ and the differential growth during wood formation by the tree can induce movements by the formation of reaction wood tissues.^3,4^ Trees and other plants can hence fulfill their biological functions and self-optimize their mechanical properties, preventing failure and securing survival. Such outstanding properties are achieved due to the intrinsic hierarchical structure of wood, which spans from the nanoscale of the cell wall to the macroscopic features of the bulk wood.^5^ Many engineering design strategies of composite materials are inspired by features of biological materials and structures. These comprise localized orientation of natural building blocks commonly embedded in biopolymer matrices, localized density gradients of reinforcements, and matrices’ compositional changes and gradients.^6^ Although the influence of various parameters is well-known, the synthetic manufacture of materials with determined functional microstructures remains challenging and is still far from the astonishing intricacy and complexity of biological materials.

The development of additive manufacturing, and especially 3D-printing technologies, made it possible to harness the design strategies, mentioned above, for the production of customized high-performance devices. With a bottom-up layer-by-layer approach, 3D-printing technologies are versatile platforms providing high geometrical freedom, allowing the production of materials and devices exhibiting complex structures and controlled microstructures.^1^ In this context, direct ink writing (DIW) has arisen as a reliable technique for the production of nanocellulose-based composites that possess spatially tailored mechanical properties.^7−9^ Especially, building blocks such as cellulose nanocrystals (CNC) present great potential as biocompatible, renewable, and functional reinforcing agents in 3D-printed parts.^10^ Acting as rheology modifiers, CNC allow suspensions to be processed by DIW, while their anisotropic rod-like shape confers a directional reinforcing effect. DIW is a filamentary technique, and it applies shear and extensional stresses to the suspension during extrusion, conferring a preferential particles’ orientation along the printing direction. This leads to a high degree of CNC alignment^8^ and provides high spatial control over the mechanical properties of the printed parts.^11^ The development of such mechanically tailored CNC composites allows the creation of lightweight and highly optimized devices for applications subject to particular load conditions. Moreover, a combination of the spatially controlled mechanical properties with a dynamic change of physical and/or chemical properties as response to an external stimulus would further improve potential functionalization for a wide range of applications spanning from biomedical fields^12^ to food packaging.^13^ Nonetheless, the development of CNC-based composites fabricated by DIW is still in its infancy, and the triggering stimuli are mainly limited to humidity. It has been shown that CNC ink formulations can benefit from the alignment during printing and the hygroscopic nature of the nanoparticles, which leads to predetermined bending and curling, imitating cell wall dynamics in wood during hydration and dehydration cycles.^14^ However, this approach has so far only been applied to soft hydrogels with Young’s moduli of a few kPa.

Besides the renewable, biocompatible nature and the reinforcing behavior of CNC, such nanoparticles are also of great interest due to their surface chemistry, which allows for facile functionalization. The large available quantity of hydroxyl groups is suitable for covalently bonding specific chemical switches that confer adaptive properties in response to defined external stimuli such as heat, pH, solvent, and light,^15^ making these particles attractive as active functional reinforcements in 3D-printed composites. Among the different external stimuli, light is particularly attractive due to its contactless nature, which allows remote actuation with minimal impact on the material and its surroundings. In addition, the actuating signal can be modulated both spatially and temporarily in order to obtain multiple responses;^16^ hence, photoresponsive CNC-based nanocomposites have already been produced for several functionalities. By grafting coumarin^17^ or benzophenone^18^ moieties on the particle’s surface, CNC composites can react to determined wavelengths and create covalent particle–matrix bonds resulting in localized photostiffening. This function was also successfully transferred to 3D-printed nanocomposites by introducing cinnamate moieties on the CNC surface and in the polymer network.^19^ Other functionalization approaches, like the modification of CNC with azobenzene^20^ photochromes, have resulted in composites films with reversible color changes when irradiated, facilitating optical storage applications. Azobenzene functionalization of other polymers has also been investigated to develop materials with different photoresponses. The use of the azobenzene *trans*–*cis* photoisomerization as switching event allowed multiple smart behaviors, such as phototriggered movements,^21^ changes in wettability,^22^ and gel–sol transitions.^23^ These examples render CNC functionalization with azobenzene attractive for the production of 3D-printed reversible multiresponsive materials.

**Figure 1:**
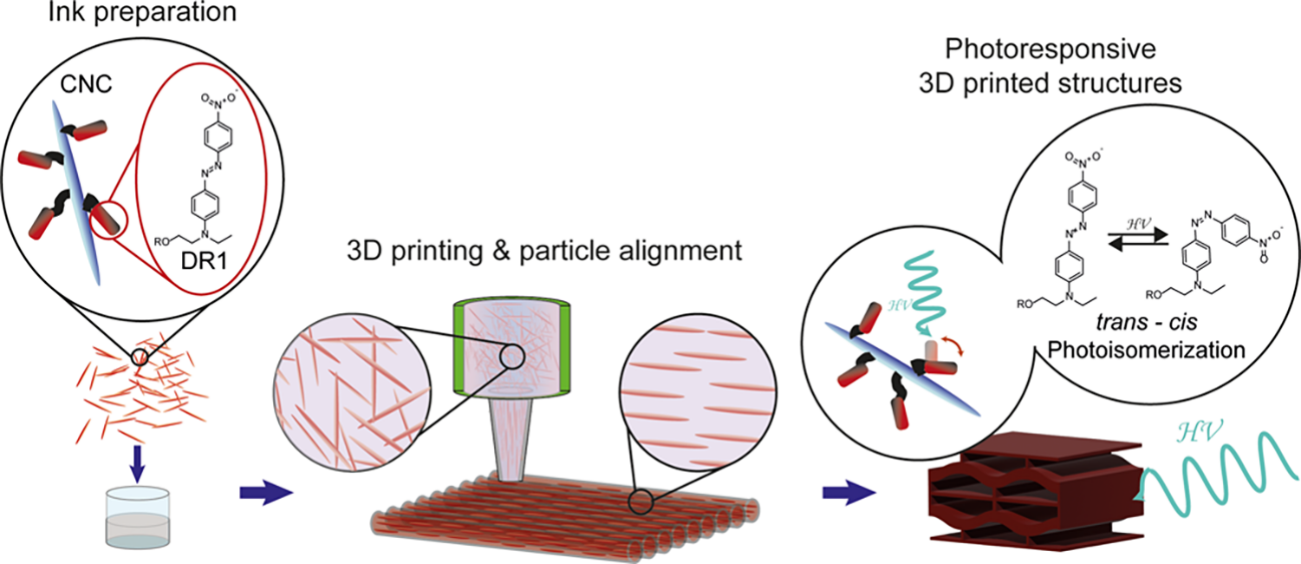
Preparation of photoresponsive 3D-printed cellulose nanocomposites. The CNC are surface-modified with Disperse Red 1 (DR1). When introduced in a polymer resin, they create a printable ink that can be processed with DIW. 3D printing locally aligns the particles along the printing direction, conferring anisotropic mechanical properties to the printed objects. The final printed structure, after curing, displays photoresponsive behaviors due to the photoisomerization of DR1.

Hence, we propose here a way to manufacture 3D complex structures presenting both fast and reversible photoresponsive behaviors and tailored mechanical properties (Figure 1). By grafting the pseudo-stilbene Disperse Red 1 (DR1)^24^ onto the surface of CNC, multipurpose active reinforcement particles that react to light with a wavelength of 475 nm can be produced. After modification, these active elements maintain their slender shape, and hence the ability to impart to Newtonian resins the rheological properties required for a successful DIW’s filamentary extrusion and particle alignment. Polymer inks can then be printed in 3D complex shapes with optimized mechanical properties while demonstrating macroscopic photoresponsive behaviors, arising from the *trans–cis–trans* photoisomerization of DR1. As a proof of concept, we dispersed these functional CNC into a polyurethane acrylate matrix and printed a composite material that can undergo reversible softening and exhibits a shape memory effect when irradiated with the appropriate wavelength. With DIW, this material can be shaped into complex 3D structures that couple mechanical behaviors, arising from their geometry, with dynamic and reversible phototriggered mechanical responses. Such combination results in devices with mechanical properties tunable on demand and suitable for applications as dynamic dampers or energy absorbers.

These renewable functionalized particles have hence a great potential for the production of diverse smart mechanical structures, i.e., structures that react to an external stimulus. By allowing the additive manufacturing of diverse matrix materials while actively conferring reversible photoresponsive behaviors, these functionalized CNC substantially increase the design freedom by adding the temporal dimension to the structure’s properties. Thus, lightweight devices for specific (dynamic) load cases can be produced and customized with fine-tuned mechanical properties that allow reacting to environmental changes.

## Results and Discussion

### Functionalization of CNC

**Figure 2:**
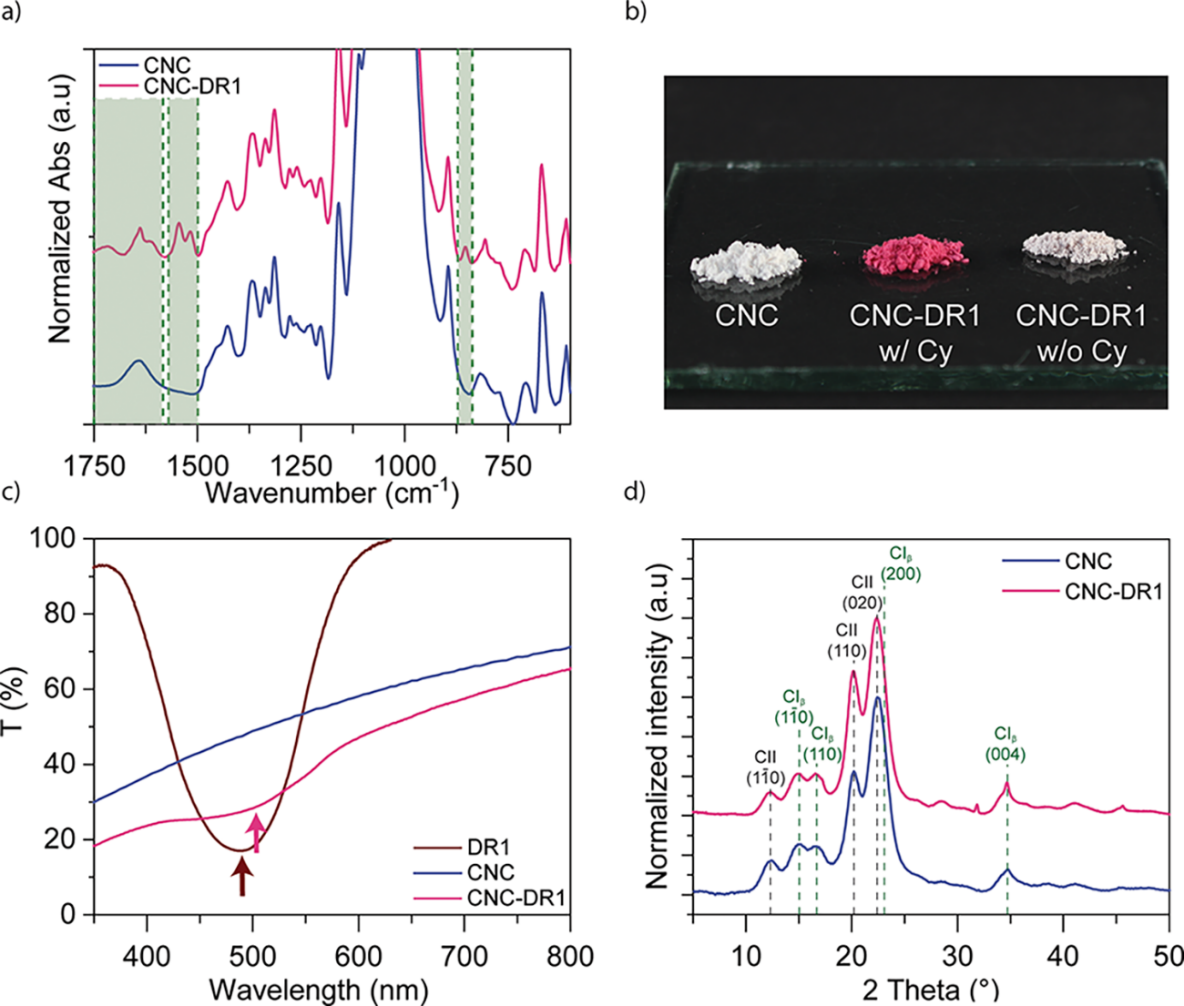
Functionalization of CNC with azobenzene molecules. (a) Enlarged FTIR spectra between 1750 and 600 cm^–1^ of CNC (before) and CNC-DR1 (after functionalization), green regions highlight the appearance of new bands. (b) Photograph comparing dried CNC (left), washed with six cycles of centrifugation and acetone renewal, dried CNC-DR1 (middle) after six washing cycles, and dried CNC that underwent the modification with DR1 without cyanuric chloride (Cy) (right) after four washing cycles. (c) UV–vis spectra of CNC with and without modification and pure DR1 in acetone, the arrows indicate the maximum absorption peaks of the spectra with the respective colors. (d) XRD patterns comparison of CNC with CNC-DR1.

We imparted functional and photoresponsive behaviors to cellulose nanocomposites by attaching DR1, an azobenzene molecule, to the surface of the CNC. To achieve such a chemical modification, cyanuric chloride was selected as a chemical linker, since the reaction undergoes two nucleophilic substitutions: one with the cellulose hydroxyl groups (−OH) and one with the DR1 ones (Figure S1) allowing covalent bonding of the azobenzene on the CNC surface.^25^ The chemical treatment could be carried out successfully, leading to strongly magenta colored CNC (Figure 2). Unmodified CNC (named CNC) and modified CNC (named CNC-DR1 for simplicity) were characterized qualitatively by ATR-FTIR and the results indicate a successful cellulose modification (Figure 2a). The appearance in the CNC-DR1 spectrum of IR bands at 854 cm^–1^ for the C–Cl bending and at 1500–1580 cm^–1^ for the C–N stretching vibrations^26^ suggests the presence of the cyanuric chloride linker. Other new bands also appear in the region 1600–1750 cm^–1^ with a prominent peak at 1636 cm^–1^, which substitutes the H–O–H vibration of adsorbed water observed in the CNC spectrum, and a second small peak at 1715 cm^–1^, both attributed to the planar stretching vibrations of the triazine ring.^27^ The sharpening of the 1636 cm^–1^ band occurred after CNC modification is assumed to occur due to the substitution of hydroxyl groups of the cellulose with the cyanuric chloride linker. The shoulder at 1610 cm^–1^ arises as a combination of triazine ring vibrations with the C=C stretching modes of the DR1 benzene rings.^28^ Indeed, comparison of CNC-DR1 spectrum with the one of CNC that reacted only with the cyanuric chloride (CNC-Cy, Figure S2) revealed that, when DR1 is employed for the reaction, the contribution of the C=C stretching makes the band at 1610 cm^–1^ appear stronger. Despite the differences observed between CNC and CNC-DR1 spectra, little differences subsist between the CNC-Cy and the CNC-DR1 IR spectra. The typical frequency bands at 1500–1550 cm^–1^ of the DR1 nitro group asymmetric stretching could not be clearly distinguished from the C–N stretching vibrations of the cyanuric chloride. To further confirm the DR1 presence on the surface of CNC-DR1, after the modification, additional proofs were qualitatively obtained by ToF SIMS, XPS, and NMR (Figure S3). All measurements revealed the presence of DR1. From solid-state NMR (Figure S3), the DR1 substitution fraction of accessible hydroxyl groups at the surface of CNC could be estimated at 0.7%, calculated according to the method employed by Xiao et al.^29^ Moreover, the substitution fraction of accessible hydroxyl on CNC-DR1 surface was estimated, considering one DR1 per triazine ring, at around 2.4%, by tracking the amount of nitrogen with elemental analysis (Table S1).

The presence of DR1 on the CNC-DR1 surface can also be observed by the naked eye (Figure 2b) since CNC-DR1 powder dried from acetone assumes a strong magenta color, which is maintained after six washing cycles of the reaction product. Differently, repeating the reaction process, for CNC with DR1 alone, without the cyanuric chloride linker, led to a whitish powder that lost almost entirely the characteristic color of DR1 already after four washing cycles. This indirectly indicates a successful grafting of DR1, mediated by the cyanuric linker, on the CNC-DR1 surface.

The CNC’s change of color is also observed in Figure 2c. Here, UV–vis spectra are illustrated comparing the transmission of 0.5 mg mL^–1^ of CNC particles (before and after modification) in acetone. Both particle suspensions show a similar behavior of increasing transmittance from 20–30% to around 60–70% with increasing wavelength. Such behavior is attributed to the light scattering observed from the two suspensions (Figure 2b). The lower transmittance of CNC-DR1 samples is due to a more homogeneous dispersion in acetone and a higher stability that promotes further scattering. Pristine CNC precipitate faster than CNC-DR1 when in acetone, resulting in lower concentrations in the light beam path during the measurement. However, the modified CNC show an absorption peak at 505 nm corresponding to the absorption region of DR1, while the unmodified particles present a constant increase in transmission as the wavelength increases. Interestingly, the maximum absorption of DR1 in acetone is at 489 nm. The redshift that occurs in the CNC-DR1 suspension arises due to a stronger electron delocalization,^30^ further indicating a successful functionalization reaction.

Despite successfully accomplishing the chemical modification, the crystalline structure of the CNC may have been altered during the process.^19^ To investigate the crystallinity in more detail, XRD was performed for both, CNC and CNC-DR1, as shown in Figure 2d. Comparing the two types of particles, it is observed that both XRD patterns are practically identical. The CNC pattern presents peaks at 14.91, 16.68, and 34.68° attributed to the crystallographic planes (11̅0), (110), and (004) of Cellulose I_β_, respectively, and peaks at 12.25, 20.15, and 22.43° attributed to the crystallographic planes (11̅0), (110), and (020) of Cellulose II.^31,32^ As previously reported by Banerjee et al.^33^ the coexistence of both Cellulose I_β_ and II polymorphs in the pristine CNC is a characteristic feature of these commercial particles. CNC-DR1 XRD pattern presents the same peaks, without shifts, nor the broadening of existing crystalline peaks of the cellulose particles that is usually attributed to a decrease of crystallite sizes.^34^ Hence, the grafting process is not detrimental to the crystalline structure of CNC, which maintains a crystallinity index of 91%, as calculated according to the method described by Park et al. “XRD peak height method”.^35^ Even though this method results in an overestimation of the crystallinity index, a relative comparison between cellulose particles seems reasonable.

**Figure 3:**
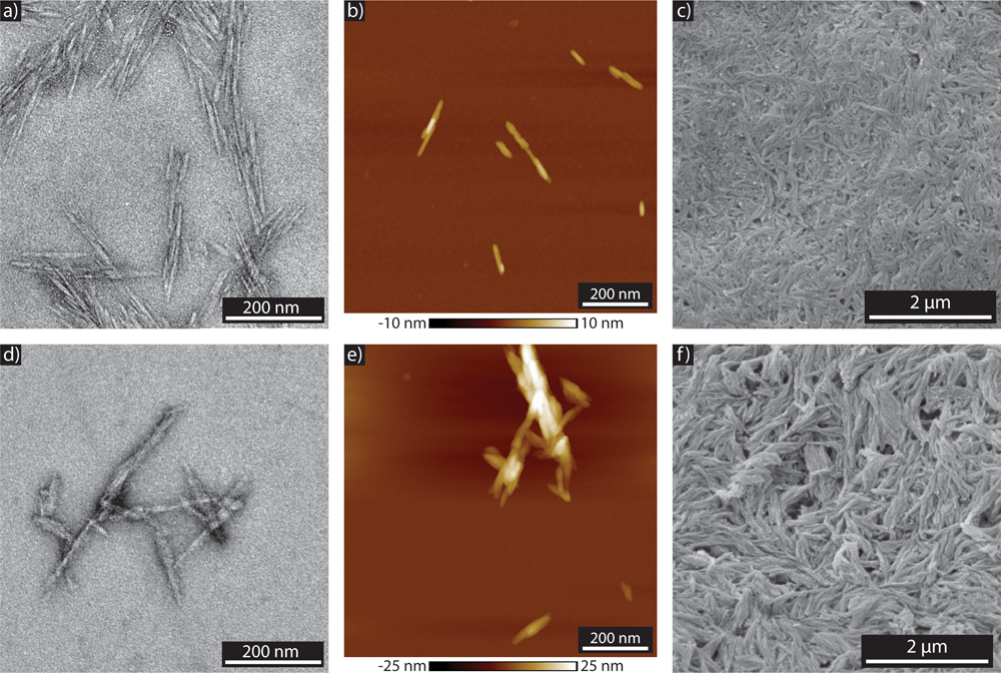
Morphological characterization of the CNC before and after the chemical reaction. (a) and (d) TEM images of CNC and CNC-DR1, respectively. (b) and (e) AFM height measurements of CNC and CNC-DR1, respectively. (c) and (f) SEM images of CNC and CNC-DR1, respectively.

After modification, CNC-DR1 must be incorporated into a polymer matrix in order to obtain a printable ink. The anisotropic shape of CNC plays an important role on the rheology of the suspension, as well as on the directional tailoring of the printed part’s mechanical properties. To ensure a strong shear thinning behavior of the ink and successful alignment of the CNC during extrusion, the aspect ratio of the CNC needs to be preserved after modification. Hence, the morphology of modified and unmodified CNC was characterized with electron and atomic force microscopy (AFM) (Figure 3). These analyses confirm that the chemical treatment of CNC does not modify the morphology of single particles. Transmission electron microscopy (TEM) and AFM observations show the morphology of both CNC (Figure 3a,b) and CNC-DR1 (Figure 3d,e) single particles. By measuring the lengths and diameters of over 60 particles of each of the two CNC types from the AFM and TEM images, it was observed that unmodified CNC possess lengths of 117.9 ± 42.1 nm and widths of 4.7 ± 1.3 nm, while CNC-DR1 show an average length of 124.3 ± 44.1 nm and widths of 4.7 ± 1.4 nm. Despite the CNC morphology not being affected by the surface modification, a slight tendency of modified particles to agglomerate was noticed. Observation of isolated CNC-DR1 single particles was more sporadic than for CNC, often resulting in images with agglomerated elements similar to Figure 3e. This may arise from the formation of covalent bonds between CNC. Unreacted Cl of the cyanuric chlorine linkers could react with the free hydroxyls of the CNC surface, thus leading to the formation of such small clusters. To have a full overview of the morphological properties of CNC, scanning electron microscopy (SEM) observations were also carried out. Dry CNC (Figure 3c) and CNC-DR1(Figure 3f) films obtained from evaporation of the CNC/acetone suspensions with the same concentration, were observed by SEM. The CNC display the presence of thin filamentary agglomerated bundles as a result of a hornification process.^36^ Similarly, CNC-DR1 also agglomerate, forming films with larger and bigger surface bundles and appear slightly less hornified.

### Direct Ink Writing of 3D Structures

**Figure 4:**
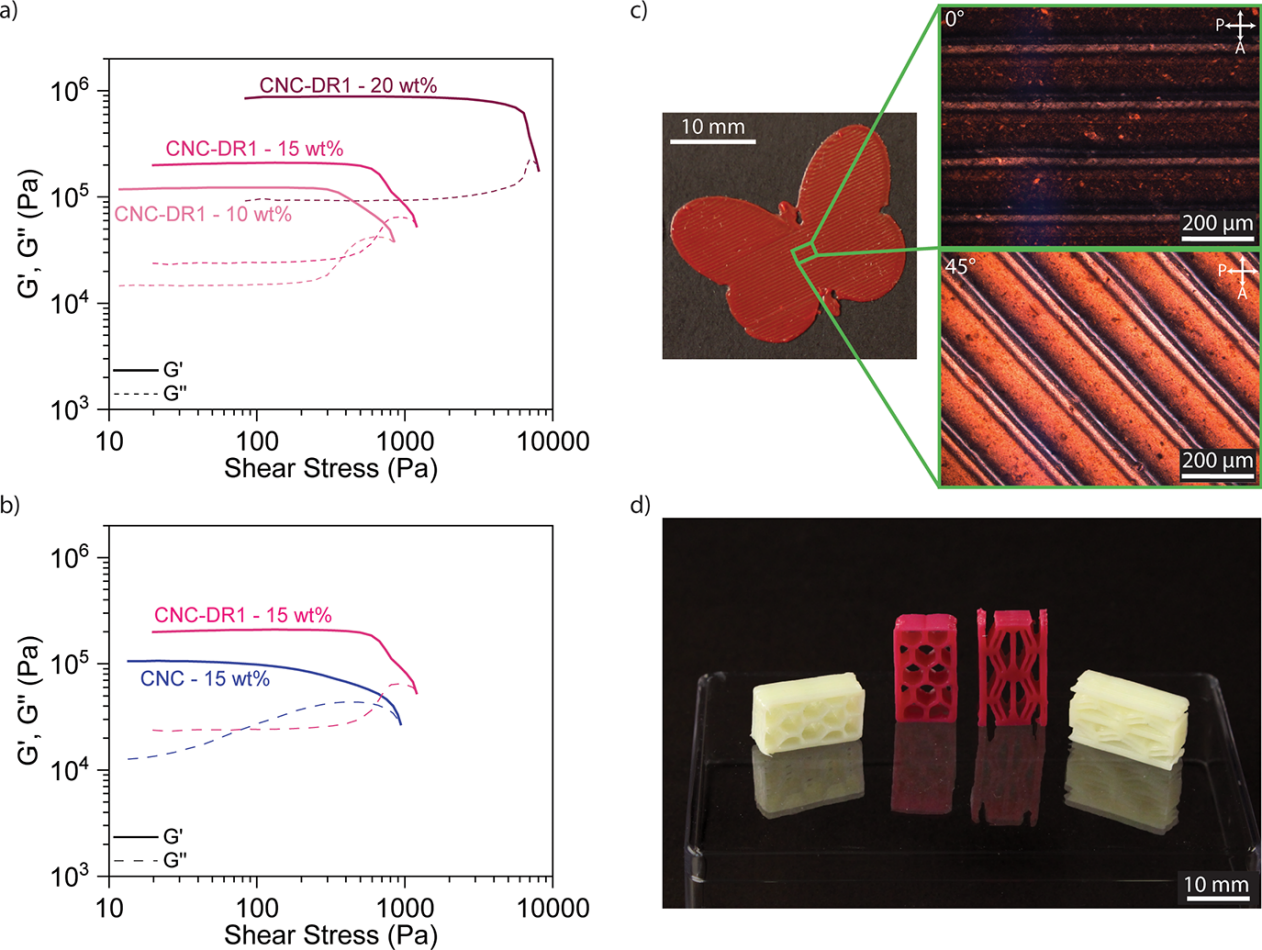
Characterization of the printable inks and manufactured demonstrators. (a) Oscillatory rheology of CNC-DR1 inks with different particle concentrations and (b) comparison of rheological behavior with unmodified CNC. (c) Butterfly composed of a 3D-printed single layer of PUA-HEMA with 15 wt % of CNC-DR1 particles. The micrographs represent polarized optical microscopy observations of the layer with printing direction at 0° and 45° angle with the polarizer. (d) 3D-printed structures with 15 wt % of either unmodified CNC (white) or functionalized particles (red), manufactured by a single step of DIW.

Modified CNC-DR1 and unmodified CNC particles were incorporated into a polyurethane acrylate (PUA) matrix composed of 49 wt % PUA, 49 wt % (hydroxyethyl)methacrylate (HEMA), and 2 wt % of photoinitiator Irgacure 819. 3D-printed composites must be manufactured by a DIW approach. Consequently, the inks need to meet specific rheological properties known as printability requirements.^9,19,37^ More precisely, inks for DIW should present a storage modulus *G′* and a loss modulus *G″* bigger than few kPa, an apparent yield stress τ_*y*_ bigger than 100 Pa while demonstrating a strong shear thinning behavior. In this work, three concentrations of CNC-DR1 were investigated by oscillatory rheology (Figure 4a), namely 10, 15, and 20 wt %. For all tested concentrations, the resulting inks feature a strong shear thinning behavior and rheological properties that fulfill the above-mentioned printability requirements. The storage and loss moduli of the tested inks display, at low shear stresses, a first plateau region where *G′* > *G″* due to the formation of a percolating network by the nanocellulose particles.^8^ The plateau region is followed by an initial drop of *G′* and an increase in *G″* at higher shear stresses, until the dynamic yield stress where *G′* = *G″* is reached, marking the transition from a solid-like response to a liquid-like behavior. Specifically, the yield stress defines the moment when the percolating network disrupts and the CNC particles align and order, inducing a strong shear thinning behavior,^38^ further confirmed with rotational rheology (Figure S4). Increasing the concentration of CNC-DR1 in the polymer matrix enhances this rheological behavior, shifting both storage and loss moduli, and the dynamic yield stress to higher values. As the inks become more concentrated in CNC-DR1, from 10 to 20 wt %, the *G′* passes from 1.2 · 10^5^ to 8.8 · 10^6^ Pa and the τ_*y*_ increases from 844 to 7750 Pa. All three concentrations of CNC-DR1 in the PUA-HEMA ink formulations meet the printability criteria. The 15 wt % ink was the concentration of choice for the DIW printer employed during this study. This concentration allows maximizing the content of CNC in the system while allowing a wider range of printing pressures to optimize the printing quality. On the contrary, an ink with 20 wt % concentration would require the maximum printing pressures of our system (ca. 5.5 bar) and it would be not possible to change this parameter.

The 15 wt % CNC-DR1 ink shows slightly higher storage modulus and yield stress compared to the rheology of the unmodified CNC ink with the equivalent particles’ concentration (Figure 4b). The ink with unmodified particles shows *G′* = 10^5^ Pa and τ_*y*_ = 938 Pa, while the CNC-DR1 ink presents *G′* = 2 · 10^5^ Pa and τ_*y*_ = 1202 Pa probably arising from the presence of slightly aggregated clusters. These could decrease the maximum packing density allowed in the ink, which would result in higher viscosities for the same volume fraction of particles.^39^ Hence, the surface chemistry of CNC-DR1 results in a stiffer ink below the yield stress, meaning that this ink is more suitable than the one with untreated CNC for printing self-standing structures. Indeed, the 15 wt % CNC-DR1 ink can easily be processed by DIW in several shapes and geometries. A custom butterfly shape (Figure 4c) was 3D-printed to investigate the shape fidelity of a single layer and control the post extrusion alignment of the cellulose particles. Comparisons of the initially designed dimensions of the butterfly’s digital model with the dimensions of the final 3D-printed demonstrator showed a high shape fidelity for features that are bigger than 1 mm. Printed samples prepared with the CNC-DR1 ink revealed a strong alignment of the particles along the printing direction, when observed with an optical microscope equipped with cross polarizer filters (Figure 4c). The well-known birefringence^40,41^ of CNC causes the strong change in intensity observed in the polarized microscopy images. CNC aligned to the light polarization (0°) allow undisturbed light transmission and its cancellation by the analyzer filter, leading to a dark image. On the other hand, when aligned at 45° to the light polarization, CNC birefringence promotes a strong light scattering, which maximizes the transmittance through the analyzer and produces the bright picture. The observed alignment of CNC-DR1 offers an interesting potential for optical applications, especially for wavelength selective polarization filter membranes. In fact, the CNC birefringence has previously been exploited for the inkjet printing of low concentrations (<2.6 wt %) sulfonated CNC suspensions, leading to materials that are promising for applications in optics as colorful polarization filters and in security printing for anticounterfeiting.^42^

Moving from printing in 2D to manufacturing complex 3D structures was easily achieved in a single printing step for inks containing 15 wt % CNC-DR1. Here, this is demonstrated with the production of two honeycomb structures (Figure 4d) with a height of 5 mm, each produced with both types of CNC reinforcement particles.

### Dynamic Response of Printed Materials and Structures

**Figure 5:**
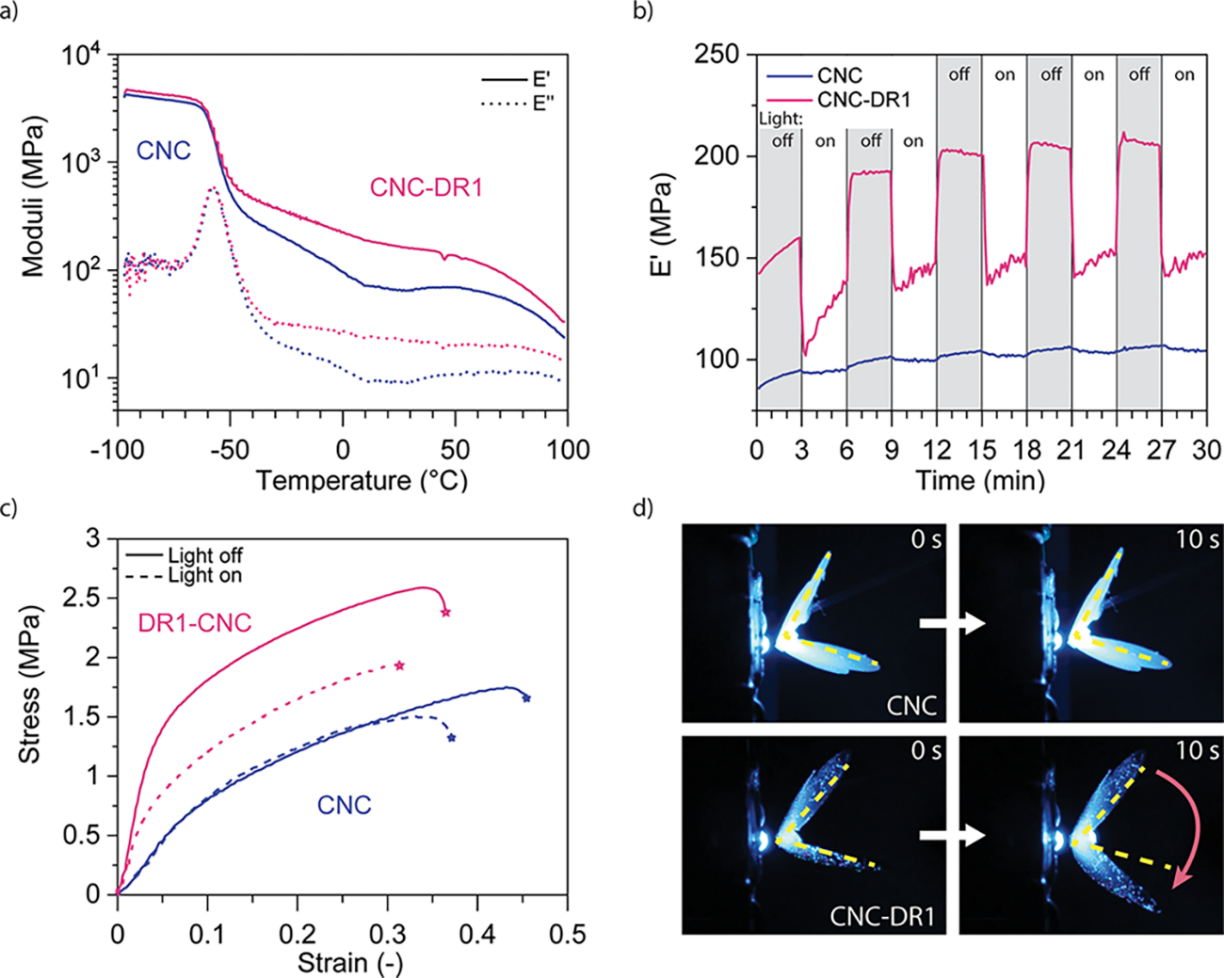
Photoresponsive properties of the 3D-printed PUA-HEMA composites. (a) Thermal DMA operated between −100 and 100 °C in tensile mode. (b) Photoresponse of the storage modulus of the composites over time. The gray regions represent measurements in dark, while white regions are measurements under illumination. (c) Tensile tests at equilibrium for samples before or during illumination. (d) Shape memory effect observed on a PUA-HEMA 15 wt % CNC-DR1 printed butterfly. The folded butterfly recovers most of its initial shape after 10 s of illumination, while a butterfly composed of PUA-HEMA 15 wt % CNC does not present such a behavior under illumination.

In order to understand both the effects of regular and photoresponsive DR1 modified CNC particles on the printed materials, the mechanical properties of 200 μm thick 3D-printed single layer composites were analyzed as a function of temperature and external illumination with an Opulent Cree LED at a wavelength of 475 nm (Figure 5). The first characterization compares CNC-DR1 with unmodified CNC printed films using thermal dynamic mechanical analysis (DMA) (Figure 5a). The comparison of 15 wt % CNC-DR1 with 15 wt % CNC composites shows that the surface modification introduced on the particles leads to stiffer materials. Both composites (CNC and CNC-DR1) have a glass transition temperature (*T*_g_) of −55 °C and the difference in storage modulus increases after the materials reach the amorphous region, before decreasing again after 100 °C. Results of differential scanning calorimetry (DSC) measurements from −100 to 200 °C (Figure S5) show that the *T*_g_ of both materials is around −59 °C, being in agreement with the results indicated by DMA measurements. Moreover, DSC analysis showed that both materials undergo melting at 123 °C with an onset temperature lying around 60 °C, thus explaining the strong final drop of storage moduli occurring after 80 °C in the DMA measurements. At 25 °C, CNC-DR1 composites demonstrate a clearly stiffer behavior with a storage modulus of 165 MPa compared to 66 MPa for unmodified CNC. The increase of the elastic properties of CNC-DR1 composites may stem from an interlocking of the CNC network, ensured by agglomerated CNC-DR1 (Figure 3e). During the reaction, cyanuric chloride groups might form covalent bonds with the cellulose’s hydroxyl groups and promote such agglomeration. Furthermore, such an effect could also arise from the introduction of azobenzene’s hard segments. In a previous work, we noticed that the presence of noncovalent bonded DR1 in liquid crystalline elastomer CNC composites promoted an increase in both, elastic modulus and strength of the materials.^43^

The photoresponsive effect of grafting the azobenzene to CNC surfaces was further studied with a DMA analysis under illumination. The printed CNC-DR1 composites display a reversible photosoftening when irradiated with light of 475 nm wavelength (Figure 5b). The dynamic softening was measured with a time sweep analysis in a DMA, during which an LED lamp (Opulent Cree, 3W) was set 5 mm away from the center of the sample. The light was then switched on and off every 3 min. These measurements show that the CNC-DR1 composites lose 32% of their storage modulus in less than 10 s after the light shines on them. This photoresponse is fully reversible. In contrast, the composites with unmodified CNC show only a slight reaction to the illumination that is attributed mainly to the heat generated from the illumination setup. The strong softening of CNC-DR1 composites arises from a photothermal effect generated by the strong light absorbance of DR1 moieties. During the illumination, DR1 molecules undergo fast *trans–cis–trans* conformational changes,^24^ generating temperatures reaching 200 °C in the near local molecular environment.^44^ For the CNC-DR1 composites, an infrared camera (Figure S6) revealed a maximum surface photogenerated temperature of 92.7 °C for the irradiated regions when the lamp is at a distance of 5 mm, confirming a strong photothermal effect of the CNC-DR1 particles. Instead, unmodified CNC composites did not show a photothermal effect, only a slight increase in temperature of 2 °C attributed to the heat irradiated from the illumination setup. The CNC-DR1 samples illuminated at 5 mm of distance show an average surface temperature around 60 °C for the whole irradiated region. This further suggests that the photosoftening arises from the increase in temperature. The storage modulus of CNC-DR1 composites, obtained by thermal DMA experiments in Figure 5a, exhibits a drop of 33% when the temperature increase from room temperature to 60 °C. Such softening is comparable to the drop in modulus observed as a response to the irradiation.

After the dynamic observation of the current photosoftening, the effect of illumination at equilibrium conditions was also studied by microtensile tests^45^ (Figure 5c), during which an LED lamp (Opulent Cree, 3W) was set 10 mm away from the center of the samples. Both unmodified CNC and CNC-DR1 composites were tested in dark and during irradiation. In dark, both composites demonstrate coherence with the DMA measurements. Unmodified CNC composites are weaker than CNC-DR1 ones, with elastic moduli measured in the dark around 10 and 30 MPa for CNC and CNC-DR1 composites, respectively. Irradiation did not lead to substantial differences in stress–strain behavior of the CNC composites compared to the samples tested in the dark, while irradiation of CNC-DR1 composites resulted in a loss in stiffness and strength. The elastic modulus of such composites strongly decreases from 30 to 16 MPa (Figure S7) while the maximum strength slightly decreases from 2.3 to 1.8 MPa when samples were illuminated. The photoresponsive softening, being fully reversible, allows CNC-DR1 composites, which were illuminated with successive intervals of irradiation, to switch between mechanical behaviors in the dark and under illumination (Figure S8).

The photogenerated heat produced from the CNC-DR1 reinforcements also proved to trigger the shape memory behavior of the PUA-HEMA matrix (Figure 5d). To clearly demonstrate this effect, we 3D-printed a butterfly using the 15 wt % CNC-DR1 ink and we folded and maintained its wings deformed until they kept an angle of 45°. Upon illumination with a 475 nm wavelength LED (Opulent Cree, 3W), the wings opened to 140° in less than 10 s, recovering 70% of the initial wing’s opening before deformation. On the contrary, when irradiating another butterfly, folded in the same manner but composed of 15 wt % CNC in the PUA-HEMA matrix, the wings do not move or open even after 60 s of illumination (Figure S9). Nonetheless, both CNC and CNC-DR1 composites react to temperature (Figure S10) and display a thermally activated shape memory behavior, confirming the actuation of DR1 by a photothermal effect. Polyurethanes are known to exhibit a shape memory effect activated by temperature.^46,47^ This phenomenon arises in thermoset polyurethanes as the PUA-HEMA matrix of the current inks, due to the presence of cross-linked hard and other soft polymer segments. The former polymer segments define the permanent shape while the latter ones are responsible for the temporary shapes and the transition temperatures.^48−50^ In particular, the PUA-HEMA matrix studied in this work demonstrates a very broad melting temperature (Figure S5) indicating that the melting of the soft segments is responsible for the shape memory behavior.

At 15 wt % concentration, CNC-DR1 particles are providing printability to the uncured PUA-HEMA matrix. An increase in the elastic modulus and strength, a strong photosoftening, and a phototriggered shape memory behavior are the major effects on the cured composites. To highlight such properties and demonstrate the advantage of printing functional materials, two types of honeycomb structures were manufactured. These honeycombs were produced by 3D printing the 15 wt % CNC-DR1 PUA-HEMA ink with the goal to combine the geometry (3D printing) with functionality (photoresponse) and to give rise to targeted mechanical responses. First, hexagonal honeycombs were manufactured as examples of energy absorbing structures.^51^ Second, we produced so-called negative stiffness structures composed of prismatic cells with two double curvature beams by DIW. The latter structures have the particularity to present energy absorption in a recoverable way.^52^ The beams are designed to allow energy dissipation as they undergo a first mode buckling deformation displaying negative stiffness during such an event. These negative stiffness honeycombs have already demonstrated the influence of a well designed geometry for energy absorption and shock isolation applications.^53,54^ With the developed DIW ink, both types of honeycomb could be easily printed and utilized to demonstrate photoresponsive dynamic energy absorbers.

**Figure 6:**
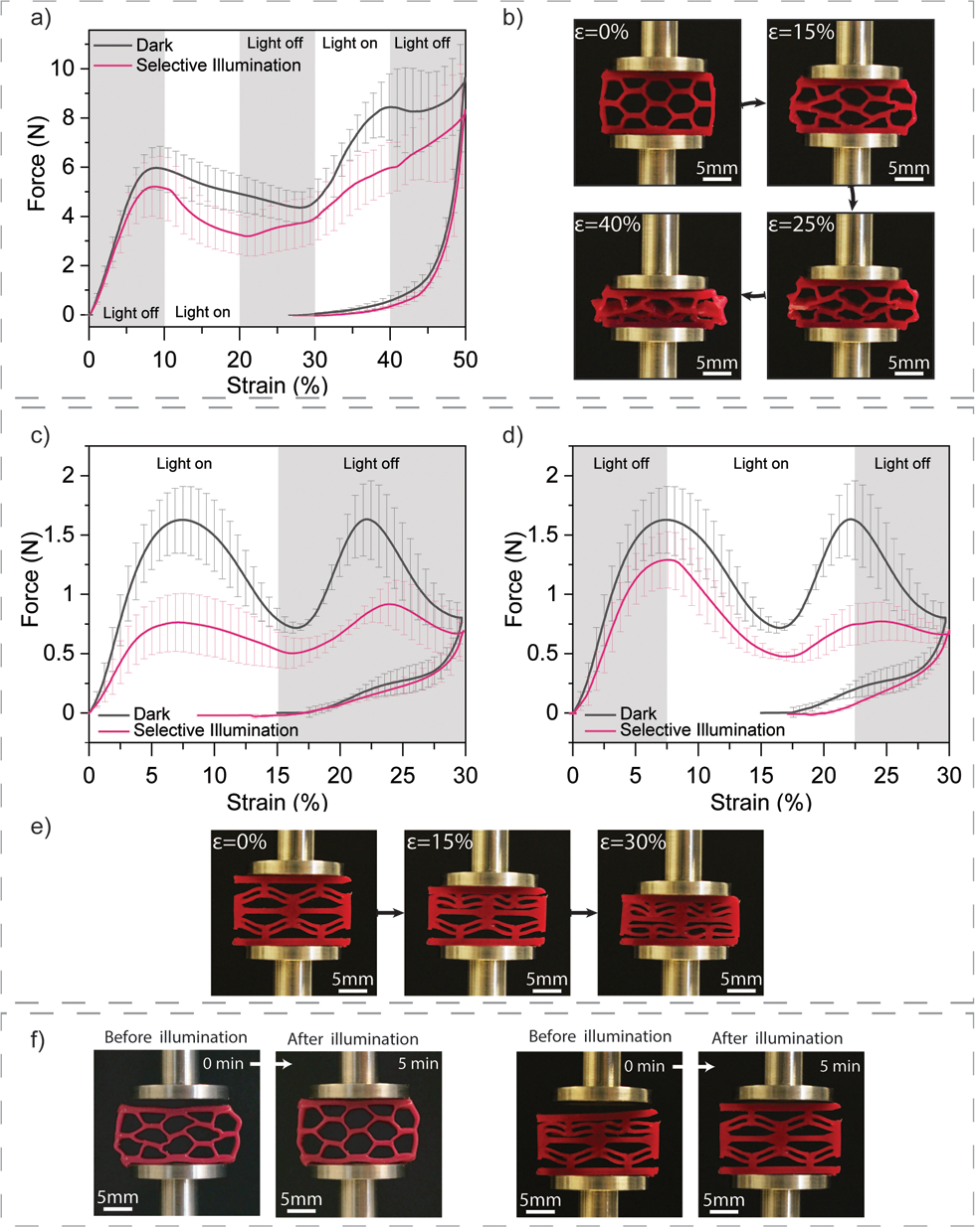
Combination of 3D-printing complexity with dynamic response to light of 3D-printed cellular structures. (a) Compression tests, in dark or under selective illumination, of hexagonal honeycombs composed of PUA-HEMA with 15 wt % CNC-DR1. For selective illumination measurements, the light was off in the gray regions, while it was switched on for the white regions of the graph. (b) Images of deformed samples under compression for different strains. (c) and (d) Compression tests in dark or under selective illumination of negative stiffness structures composed of PUA-HEMA with 15 wt % CNC-DR1; for selective illumination measurements, (c and d) represent different illumination profiles, the light was off in the gray regions and it was on in the white regions of the graphs. (e) Deformation under compression of a tested negative stiffness structure. (f) Shape memory effect of compressed hexagonal honeycombs and negative stiffness structures after 5 min of illumination, both structures are composed of PUA-HEMA with 15 wt % CNC-DR1.

Once the structures were printed, we carried out quasi static compression tests to characterize energy absorption features of the 3D-printed structures while illuminating them at selected deformations (Figure 6). This produces a distinct response to the compressive force, allowing to tune the quantity of energy absorbed by a defined structure. Indeed, the hexagonal honeycombs show a clear difference in their energy absorption capability when comparing samples compressed in the dark with samples compressed upon selective illumination (Figure 6a). The structures were compressed until 50% of their original size and released. The black curve represents the averaged mechanical behavior of the structures in the dark while the magenta curve shows what happens when the light irradiates the samples every 10% of strain intervals. In the dark, the hexagonal structure deforms linearly, before having a negative slope from 10% to 30%. After 30% of compressive strain, a second linear increase in force is observed due to the connection of the deformed cell walls, as can be observed in Figure 6b, occurring after 25% strain. When the functional composite structure undergoes irradiation intervals, the reversible photosoftening quickly arises under illumination. This generates a dynamic weakening of the structure that can be observed by the fast change of force-strain slope at 10% followed by a prompt strengthening at 20% strain, after stopping the irradiation. In the further course of the test, the illumination at 30% strain produces again a strong change in the mechanical properties of the composites. The slope of the force decreases from the nonilluminated condition and produces a continuous increase of force until the end of the cycle. Interestingly, both conditions led to the same result during unloading, the structures could recover only until 25% of compressive deformation.

The negative stiffness structures that were manufactured with 15 wt % of CNC-DR1 ink were loaded in the same compression experiments as the hexagonal honeycombs, but until 30% of compressive strain and different illumination profiles (Figure 6c and d). In the dark, the printed structures show a mechanical behavior with two maximum peaks of force around 1.5 N, representing the snapping of the double curvature beams of the prismatic cells (Figure 6e). Contrary to literature,^52,55^ the composite structure does not seem to be recoverable, since during the unloading, it can recover only until 10–15% of strain with the slight presence of a snapping peak. Despite the fact that the process parameters have been optimized for both high resolution and CNC alignment (e.g., 250 μm nozzles diameter, 3.5 bar of pressure at 10 mm s^–1^), unnoticeable defects might occur during printing of sharp corners, which could create local stress concentrations and lead to the formation of plastically deformed regions. Nonetheless, light irradiation provides important changes to the compressive behavior of the negative stiffness honeycombs. When irradiated from the beginning of the compressive loading (Figure 6c) to half of the deformation, the photosoftening completely weakens the negative stiffness structures, leading to a broad initial force peak (reaching 0.75 N) followed by a second peak in the dark where the mechanical resistance of the structures can only slightly recover from the initial illumination (0.90 N). On the contrary, when the illumination occurs at the maximum of the first force peak (Figure 6d) the softening of the negative stiffness structures leads to a marked change of slope followed by a strong decrease of the second force peak, which reaches again 0.75 N. During unloading the second double curvature cell does not snap back to its original position and remains blocked in its compressed state. This is also seen from the graph in Figure 6d where the recovery force peak is leveled out.

Certainly, both types of structures can benefit from the shape memory behavior previously observed, and recover their initial shape. After carrying out the compression, 5 min of illumination trigger the shape recovery of the tested hexagonal honeycomb and negative stiffness structures (Figure 6f). For the negative stiffness honeycomb, the recovery of the initial shape could be carried out three times. After the first shape recovery, the negative stiffness structure undergoes a slight decrease in the force threshold of the buckling deformation, decreasing from 1.6 to 1.3 N. However, the compression of the same part could be repeated two more times with minor shifts of the force peaks (Figure S11), arising from buckling instabilities, but maintaining a similar resistance with force peaks varying between 1.15 and 1.3 N. Finally, the introduction of CNC-DR1 in the PU matrix allows for the material to be additively manufactured and confers both mechanical reinforcement as well as photoresponsive softening. Moreover, such material demonstrates to be suitable for the production of dynamic energy absorbers by 3D printing, which present customizability of the mechanical response and, interestingly, also improvement of recoverability of the compressed structures. Nevertheless, the greatest advantage of the modified CNC (CNC-DR1) lies in the possibility to be dispersed in other polymer matrices to exploit the photothermal effect for shape changing purposes, as could be the case for PNIPAM hydrogels,^15^ or to directly exploit the conformational changes of DR1 to trigger liquid crystalline elastomer shapemorphing.^16^

## Conclusion

We demonstrated an approach that allows the fabrication of composite materials with DIW while conferring photoresponsive behaviors to the obtained complex 3D-printed structures. Among the different photoresponses available by grafting DR1 azobenzenes on CNC, we harnessed the heat generated by the rapid *trans–cis–trans* photoisomerization to produce dynamic energy absorbers that can be functionally tuned by illumination, both spatially and temporally. The DR1 surface grafting on renewable CNC was confirmed by FTIR, NMR, XPS and UV–vis. The chemical modification led to a change in color of the nanoparticles without modifying the crystalline structure or the morphology of the particles while maintaining their anisotropic shape. The modified nanoparticles could be introduced into a PUA matrix, strongly influencing its rheology and promoting the formation of gel-like inks that fulfill printability requirements, thus allowing the processing of 3D parts by DIW. The strong photothermal effect confers photosoftening ability and a shape memory effect to the 3D-printed structures. The strength of this approach lies in combining such photoresponses with the DIW ability to create 3D complex shapes, as honeycombs and negative stiffness structures, presenting local alignment of CNC. As a result, such devices could be printed in a single step and their mechanical behavior under compression forces could be tailored by temporally illuminating the structures during loading. The material produced in this work presents an interesting potential for the production of shock and vibration protection or dynamic energy absorbing devices that are operated in specific load conditions, for instance, as in prosthetics or in soft robotics. This demonstrates that adding functional responses to 3D-printed devices enhances the design freedom and adaptability of lightweight mechanically tailored structures. However, the potential of our CNC is not limited to the produced material, since CNC-DR1 would allow exploiting the azobenzene *trans–cis–trans* photoisomerization in other compatible polymer matrices. This could allow for 3D printing of devices with different photoresponses. By introducing such nanoparticles into liquid crystalline elastomers, the photomechanical effect would lead to dynamic and reversible shape changes. For instance, the use of a matrix including cyclodextrins in the polymer network would lead to gel–sol transitions. Furthermore, the development of these multipurpose active CNC enables to transfer material technologies that were formerly limited to 2D shape into 3D complex structures, in which geometrical effects, spatially tailored mechanical properties and photoresponses are combined. With this technology, highly customized devices with outstanding functional properties can be produced based on renewable materials, moving a step further toward independence from fossil fuel resources. Finally, by allowing and improving emulation of biological materials’ structures and properties, the developed devices could also contribute to deepening the understanding of natural materials and their complexity.

## Experimental Methods

### Materials

Cellulose nanocrystals (CNC) obtained from sulfuric acid hydrolysis of eucalyptus pulp were supplied by University of Maine (United States). The reagents Disperse Red 1 (DR1) (95%) and Cyanuric Chloride (≥98%) were acquired from Sigma-Aldrich and Fluka Chemie GmbH, respectively. The catalyst 2,4,6-trimethylpyridine was supplied by VWR (Acros Organics). The polymer matrix was composed of Polyurethane Acrylate (BR3741A) kindly supplied by Dymax and 2-hydroxyethyl methacrylate (HEMA) acquired from Sigma-Aldrich. The photoinitiator bis(2,4,6-trimethylbenzoyl)-phenylphosphineoxide (IRGACURE 819) was obtained from BASF. Finally, dimethyl sulfoxide (≥99.5%) was acquired by Sigma-Aldrich and used without further purification.

### Functionalization of CNC

In order to graft the azobenzenes on the surface of the CNC, 1.5 g of DR1 were added into a 3-necks round-bottom flask containing a suspension of 3 g of CNC in 150 g of acetone. Four g of cyanuric chloride were successively dissolved in 30 g of acetone and added slowly to the cellulose suspension. Then, 2,4,6-trimethylpyridine (3.28 mL) was slowly poured in the suspension. The dispersion was continuously stirred and heated up to 55 °C in a nitrogen atmosphere for 15 h. The glass reactor was equipped with a condenser to avoid evaporation of the acetone. The reaction was stopped by quenching the suspension in an ice bath. The modified CNC were purified by 6 centrifugation cycles (Rotina-380, Hettich) at 5000 rpm for 5 min with renewal of the solvent. The solid content of the final suspension was determined by solvent evaporation.

### Preparation of the Inks

The inks that were investigated in this work contained 10, 15, or 20 wt % CNC (modified or unmodified) and the polyurethane-HEMA matrix. The matrix components were mixed with 49 wt % of the difunctional polyurethane acrylate oligomer (PUA), 49 wt % 2-hydroxyethyl methacrylate monomer (HEMA), and 2 wt % bis(2,4,6.trimethylbenzoyl)-phenylphosphineoxide (Irgacure 819) as photoinitiator. After mixing of the matrix components, the desired amounts of CNC were added as an acetone dispersion. The solvent was successively evaporated overnight, while continuously stirring the mixture at 40 °C. After this step, the ink was mixed 3 times in a SpeedMixer (DAC 150.1FVZ), with cycles composed of 1 min at 1000 rpm, 2 min at 1500 rpm, and 4 min at 2000 rpm. After mixing, the ink was transferred to a 30 mL UV shielding cartridge. The cartridge was centrifuged for 10 min at 3000 rpm to remove any air bubbles from the ink.

### 3D Printing of Structures

3D structures were printed using a conical nozzle with an inner diameter of 0.25 mm. The prepared cartridges were mounted on a direct ink writer (DIW) printer (3D-Bioplotter “Manufacturer Series”, EnvisionTEC, Germany). All inks containing modified or unmodified CNC, were extruded with a printing speed of 10 mm/s and a pneumatic pressure ranging between 1 and 4 bar onto a previously hydrophobized glass slide. Each layer was illuminated for 10 s with three monochromatic LEDs (365 nm wavelength, 3 W) in air. The printed final structures were cured for 10 min in a nitrogen atmosphere using a custom-made illumination setup composed of 5 monochromatic LEDs (365 nm wavelength, 3 W).

### Shape Memory Observation

The unfolding of the wings of a 3D-printed butterfly was characterized as follows: the wings of the butterfly were manually folded closed and maintained until no elastic response could be observed. Then, the materials were illuminated at a distance of 5 mm from an LED light (475 nm wavelength, 3 W). The movement of the demonstrator while illuminated was captured with a camera (EOS 100D, Canon) with an aperture of 9.0 and an exposure time of 100 ms.

### Optical Microscopy

The images of the printed structures were taken with an optical microscope (Zeiss, Axioplan) equipped with a digital camera (Leica DFC 420). The microscope was also equipped with cross polarizer filters and the samples were rotated from 0° with respect to the polarizer to 45°.

### Fourier-Transform Infrared Spectroscopy (FT-IR)

The functionalization reactions were investigated with an FT-IR spectroscope (Tensor 27 IR, Bruker) equipped with a ZnSe crystal in attenuated total reflectance (ATR) mode. The samples were prepared by drying the suspensions for 48 h in a 60 °C oven to dry and evaporate the solvent. The FT-IR spectra were recorded from 4000 to 600 cm^–1^ with 64 scans per sample and a resolution of 4 cm^–1^. Three spectra were recorded per sample; the spectra were baselined and averaged with OPUS scope software. Finally, each spectra was normalized by its maximum value to allow visualization and comparison of multiple spectra.

### Ultraviolet–Visible (UV–Vis) Spectroscopy

Quartz glass cuvettes were filled with a DR1 solution with 10 μg mL^–1^ in acetone for the analysis of the pure azobenzene. Quartz cuvette containing 0.5 mg mL^–1^ of CNC (either unmodified or modified) in acetone. The cuvettes were shaken thoroughly before measuring the samples with an UV–vis spectrometer (Cary 1E, Varian). The spectra were recorded between 800 and 200 nm with a step size of 5 nm and the measurements were repeated 3 times for each sample. The representative measurements are illustrated in the manuscript.

### Powder X-ray Diffraction (XRD)

Diffraction graphs were measured using an XRD system (Panalytical, X’pert Pro) equipped with Cu radiation (Kα_1_ = 1.54056 Å and Kα_2_ = 1.54439 Å). The samples were ground and placed on a zero-background silicon sample holder. The diffraction data was measured from 5 to 80 degrees with a step size of 0.026 degrees. The sample was rotated at a speed of 8 s per revolution during the measurement to increase the sampling statistic. The curves were baselined and plotted for calculating the crystallinity index. The crystallinity index was determined based on the intensity method described by Park et al.^35^ In this method, I_002_ peak intensity located at a 2 θ angle of around 22.5° and the amorphous peak intensity I_AM_ at approximately 18° are measured. The crystallinity index was calculated according to following eq 1:
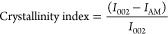
1

### Atomic Force Microscopy Scanning (AFM)

Single particles were imaged with the Icon3 AFM (Bruker) in soft tapping mode using silicon tips (RTESPA-150, Bruker). Later, the images were flattened, and artifacts were removed with NanoScope analysis software from Bruker. The samples preparation for the AFM analysis was conducted based on the process described by Arcari et al.^56^ Briefly, the modified and unmodified CNC were dispersed in Milli-Q water to reach a final concentration of 2 mg L^–1^. Freshly cleaved mica was attached with double-sided tape to a glass slide. Twenty μL of an acqueous solution containing 0.05 vol % of (3-aminopropyl) triethoxysilane (APTES) were deposited on the mica to achieve a positive surface charge. After 60 s the mica was rinsed thoroughly with Milli-Q water and dried with a pressurized air gun. Successively, the mica was covered by the 2 mg/L CNC suspensions. After 30 s the mica was rinsed again thoroughly with Milli-Q water and dried with pressurized air. The samples were kept under vacuum in a desiccator before the measurements to prevent any contamination. Measurements of diameters of CNC were based on the vertical cantilever displacement, while length were extracted from the images with the NanoScope analysis software.

### Scanning Electron Microscopy (SEM)

The cellulose nanoparticles were imaged with a NanoSEM 230 (FEI) SEM at a distance of 10 mm and an accelerating voltage of 5 kV. The samples were prepared by evaporating the acetone from the suspension in a 60 °C oven. The dried powders were sprinkled on a carbon tape stuck to aluminum sample holders. A 7 nm layer of platinum was sputtered on the samples as conductive layer. The samples were stored under vacuum in a desiccator before imaging.

### Transmission Electron Microscopy (TEM)

The modified and unmodified CNC were imaged with a JEM-2200FS (Jeol) TEM and an acceleration voltage of 200 kV. Samples were prepared and deposited on carbon coated-grids. The carbon-coated grid surface was made more hydrophilic by 10 s of oxygen plasma treatment. A 30 μL drop of 2 mg/L CNC water suspensions was deposited on Parafilm and the grid was laid on top of the drop for 30 s. Then, the grid was placed in a drop of 2 wt % of uranyl acetate for 30 s to stain the samples. The samples were dried and kept under vacuum in a desiccator before imaging. Analysis of lengths of CNC was conducted with the help of ImageJ software.

### Nuclear Magnetic Resonance (NMR)

The measurements were carried out on an AVIII HD 400 MHz wide-bore (Bruker) NMR spectrometer. For the liquid-state NMR, 30 mg of DR1 was weighed in a vial and 0.5 mL of deuterated chloroform was added to the vial. The solution was transferred to an NMR tube with a pipet. The solid-state cross-polarization magic angle spinning carbon-13 (^13^C CP-MAS) NMR spectra were acquired between 400.2 and 100.6 MHz on an Avance III 400 MHz (Bruker) spectrometer at room temperature with a 4 mm MAS probe operating at a spinning speed of 10 kHz. Approximately 50–80 mg of the sample material were densely packed into 4 mm zirconia rotors. For the ^13^C CP-MAS NMR experiments a 3.5 μs excitation pulse at 90° was applied on the ^1^H channel. Further, the contact time was set to 1 ms (optimized for best signal-to-noise) with a ramp from 100 to 50% of the power level on the proton channel. During acquisition, 3 s recycle delays, and 71 kHz SPINAL 64 proton decoupling were applied. Appropriate numbers of 1500 to 16000 scans were recorded to yield reasonable signal-to-noise ratios.

### Time of Flight Secondary Ion Mass Spectrometry (ToF SIMS)

CNC films dried from an acetone suspension for both the unmodified and DR1 modified CNC were fixed on to the ToF-SIMS plate with aluminum tape and inserted into the vacuum chamber of the ToF-SIMS.5 from IONTOF GmbH, Germany. Depth profiling was carried out using a cesium ion sputter gun with an energy of 500 eV. An area of 500 × 500 μm was sputtered and an area of 50 × 50 μm^2^ or 100 × 100 μm^2^ was measured. Bi^3+^ ions at an energy of 25 keV and a cycle time of 100 μs were used in the analysis beam. Images were measured with a resolution of 256 × 256 pixels per measurement. A floodgun was used to counteract charging at the surface and the charge compensation was automatically adjusted for each sample.

### X-ray Photoelectron Spectroscopy (XPS)

The samples were prepared as dry film from slow evaporation of acetone/CNC or acetone/CNC-DR1 suspensions. The XPS spectra were acquired with a PHI Quantum spectrometer, equipped with an Al Kα monochromatic source (1486.7 eV), a hemispherical capacitor electron-energy analyzer, and a 16–channel plate detector. All the spectra were acquired with an emission angle of 45°, in fixed analyzer transmission mode, using a nominal X-ray beam-spot size of 150 μm. Given the insulating nature of the samples, all the acquisitions were carried out under dual-beam charge compensation. The peak maximum of the C 1s was used for BE referencing. Specifically, the BE value of the alkoxy group in cellulose^57^ (2876.7 eV) was taken as reference value.

Nitrogen (N 1s) spectra were acquired at a pass energy of 23.5 eV, step size of 0.1 eV and duration of ca. 36 min while the X-ray beam was scanned over a 500 × 1000 μm^2^ area during spectra acquisition, rather than focusing on a stationary point to limit X-ray induced degradation of nitro groups. Surveys spectra were acquired on previously not irradiated areas for a duration of ca. 15 min and used to carry out elemental quantitative analysis, according to the formula in eq 2:
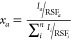
2*x*_*a*_ is the atomic concentration of the element “*a*”, and *I_i_* is the intensity (area) of the selected peak of the element “*i*”. RSFi is the associated relative sensitivity factor. The RSFs used in this work were derived from the analysis software of the XP-spectrometer. It should be noted that the approach based on eq 2 is strictly valid for materials that are homogeneous to a depth greater than the information depth of the technique.

### Rheology

The rheological behavior of the printable inks was characterized with an MCR 301 (Anton Paar) rheometer at 20 °C. The measurements were carried out with a plate–plate geometry, using a disposable aluminum plate with a diameter of 25 mm and a gap of 0.5 mm between the plates. The storage (*G′*) and loss (*G″*) modulus were determined by applying an oscillatory deformation between 0.01% and 1000% strain with a frequency of 1 Hz and a logarithmic sweep. Flow properties were measured with rotational increase of the shear rate in a logarithmic sweep. Three measurements per ink were performed and the representative measurement is reported in this work.

### Differential Scanning Calorimetry (DSC)

The thermal behavior of the composite materials was investigated using a DSC 8000 DSC by PerkinElmer with two heating cycles from −100 to 200 °C at a rate of 20 °C/min and one cooling cycle from 200 °C to −100 °C at a rate of 20 °C/min. Approximately 18 mg of the samples were placed in a pan and sealed tightly. All the measurements were carried out under nitrogen atmosphere.

### Dynamic Mechanical Analysis (DMA)

The mechanical properties of the printed composites were obtained with a DMA RSA 3 analyzer from TA Instruments. Printed films with a width of 3 mm and a thickness of approximately 200 μm were mounted with the printing direction parallel to the tensile direction at a clamp distance of 20 mm and a prestrain of 20 g. The temperature sweeps were conducted starting from −100 to 100 °C at a rate of 3 °C/min with a strain of 0.04% and a frequency of 1 Hz, using a liquid nitrogen cooler. Light illumination tests were performed at room temperature (25 °C) by placing one LED lamp (475 nm, 3 W) at a distance of 5 mm from the center of the sample. Here, a time sweep with 0.04% of strain and a frequency of 1 Hz were applied. The LED lamp was switched on and off every 3 min until the end of the measurement, after 30 min. Three samples per condition were tested and the representative one is illustrated.

### Microtensile Tests

Tensile tests on the printed composites were carried out in a custom test setup reported by Burgert et al.^45^ In summary, a step motor on a linear table allowed to vary the speed from 1 μm s^–1^ to 1000 μm s^–1^ while the loading is measured with a 5 N load cell. The samples, films of 1 mm width and a thickness of 200 μm, were glued with a cyanoacrylate glue from both sides onto rectangular plastic foils containing three holes in the centers and one for each extremity. The holders were placed onto the moving table and fixed with pin holes at the extremities. In order to not measure the foil properties, the three holes were cut. The samples were stretched at a speed of 30 μm s^–1^. To calculate the sample’s strain, the displacement of the table was divided by the sample’s length between the glued points of the sample (6 to 9 mm). Both diameters and initial length were observed and recorded with an optical microscope. For measurements under illumination, a support for the Opulent Cree LED (480 nm, 3W) was 3D-printed with a Prusa imk3 FDM in PLA and adapted to the set up in order to have a 10 mm distance from the samples. Five samples were tested for each test and sample’s condition. All samples were tested with the printing direction parallel to the loading direction. The representative measurements are illustrated.

### Compression Tests

Compression Force-strain curves of the printed structures were obtained on a RSA3 3 analyzer from TA Instruments. The 2.5 mm thick hexagonal and negative stiffness honeycombs of 15 wt % CNC-DR1 were placed in the instrument in compression mode, using circular clamps with a diameter of 15 mm. The instrument was set to operate at a speed of 30 μm s^–1^ and at a compression of 50% and 30% strain for hexagonal and negative stiffness structures, respectively. The illumination was applied by the mean of two Opulent Cree LED (475 nm, 3 W) placed at 10 mm from the samples on each side (back and front). Five samples per condition where tested. The averaged measurements are illustrated with the standard deviation.

## Supporting Information Available

Schematic representation of DR1 grafting. FTIR, ToF SIMS, XPS, solid-state NMR, elemental analysis of CNC and modified CNC, rotational rheology of CNC and CNC-DR1 inks, thermal imaging, tensile tests, shape memory effect and cyclic compression tests of 3D-printed composites (PDF)

## References

[ref1] Studart A. R. Additive Manufacturing of Biologically-Inspired Materials. Chem. Soc. Rev. 2016, 45, 359–376. 10.1039/C5CS00836K. 26750617

[ref2] Kasal B. WOOD FORMATION AND PROPERTIES | Mechanical Properties of Wood. In Encyclopedia of Forest Sciences; Elsevier: Oxford, 2004; pp 1815–1828.

[ref3] Martone P. T.; Boller M.; Burgert I.; Dumais J.; Edwards J.; MacH K.; Rowe N.; Rueggeberg M.; Seidel R.; Speck T. Mechanics without Muscle: Biomechanical Inspiration from the Plant World. Integr. Comp. Biol. 2010, 50, 888–907. 10.1093/icb/icq122. 21558248

[ref4] Clair B.; Thibaut B. Physical and Mechanical Properties of Reaction Wood. Springer Ser. Wood Sci. 2014, 171–200. 10.1007/978-3-642-10814-3_6.

[ref5] Chen C.; Kuang Y.; Zhu S.; Burgert I.; Keplinger T.; Gong A.; Li T.; Berglund L.; Eichhorn S. J.; Hu L. Structure–Property–Function Relationships of Natural and Engineered Wood. Nat. Rev. Mater. 2020, 5, 642–666. 10.1038/s41578-020-0195-z.

[ref6] Bar-On B.; Barth F. G.; Fratzl P.; Politi Y. Multiscale Structural Gradients Enhance the Biomechanical Functionality of the Spider Fang. Nat. Commun. 2014, 5, 1–8. 10.1038/ncomms4894. PMC4050259 24866935

[ref7] Kokkinis D.; Schaffner M.; Studart A. R. Multimaterial Magnetically Assisted 3D Printing of Composite Materials. Nat. Commun. 2015, 6, 8643 10.1038/ncomms9643. 26494528 PMC4639895

[ref8] Hausmann M. K.; Rühs P. A.; Siqueira G.; Läuger J.; Libanori R.; Zimmermann T.; Studart A. R. Dynamics of Cellulose Nanocrystal Alignment during 3D Printing. ACS Nano 2018, 12, 6926–6937. 10.1021/acsnano.8b02366. 29975510

[ref9] Siqueira G.; Kokkinis D.; Libanori R.; Hausmann M. K.; Gladman A. S.; Neels A.; Tingaut P.; Zimmermann T.; Lewis J. A.; Studart A. R. Cellulose Nanocrystal Inks for 3D Printing of Textured Cellular Architectures. Adv. Funct. Mater. 2017, 27, 1604619 10.1002/adfm.201604619.

[ref10] Gauss C.; Pickering K. L.; Muthe L. P. The Use of Cellulose in Bio-Derived Formulations for 3D/4D Printing: A Review. Compos., Part C: Open Access 2021, 4, 100113 10.1016/j.jcomc.2021.100113.

[ref11] Hausmann M. K.; Siqueira G.; Libanori R.; Kokkinis D.; Neels A.; Zimmermann T.; Studart A. R. Complex-Shaped Cellulose Composites Made by Wet Densification of 3D Printed Scaffolds. Adv. Funct. Mater. 2020, 30, 1904127 10.1002/adfm.201904127.

[ref12] Kirillova A.; Ionov L. Shape-Changing Polymers for Biomedical Applications. J. Mater. Chem. B 2019, 7, 1597–1624. 10.1039/C8TB02579G. 32254904

[ref13] Yu H.; Yan C.; Yao J. Fully Biodegradable Food Packaging Materials Based on Functionalized Cellulose Nanocrystals/Poly(3-Hydroxybutyrate-Co-3-Hydroxyvalerate) Nanocomposites. RSC Adv. 2014, 4, 59792–59802. 10.1039/C4RA12691B.

[ref14] Fourmann O.; Hausmann M. K.; Neels A.; Schubert M.; Nyström G.; Zimmermann T.; Siqueira G. 3D Printing of Shape-Morphing and Antibacterial Anisotropic Nanocellulose Hydrogels. Carbohydr. Polym. 2021, 259, 117716 10.1016/j.carbpol.2021.117716. 33673992

[ref15] Nasseri R.; Deutschman C. P.; Han L.; Pope M. A.; Tam K. C. Cellulose Nanocrystals in Smart and Stimuli-Responsive Materials: A Review. Mater. Today Adv. 2020, 5, 100055 10.1016/j.mtadv.2020.100055.

[ref16] Palagi S.; Mark A. G.; Reigh S. Y.; Melde K.; Qiu T.; Zeng H.; Parmeggiani C.; Martella D.; Sanchez-Castillo A.; Kapernaum N.; Giesselmann F.; Wiersma D. S.; Lauga E.; Fischer P. Structured Light Enables Biomimetic Swimming and Versatile Locomotion of Photoresponsive Soft Microrobots. Nat. Mater. 2016, 15, 647–653. 10.1038/nmat4569. 26878315

[ref17] Biyani M. V.; Weder C.; Foster E. J. Photoswitchable Nanocomposites Made from Coumarin-Functionalized Cellulose Nanocrystals. Polym. Chem. 2014, 5, 5501–5508. 10.1039/C4PY00486H.

[ref18] Biyani M. V.; Jorfi M.; Weder C.; Foster E. J. Light-Stimulated Mechanically Switchable, Photopatternable Cellulose Nanocomposites. Polym. Chem. 2014, 5, 5716–5724. 10.1039/C4PY00487F.

[ref19] Müller L. A. E.; Zimmermann T.; Nyström G.; Burgert I.; Siqueira G. Mechanical Properties Tailoring of 3D Printed Photoresponsive Nanocellulose Composites. Adv. Funct. Mater. 2020, 30, 2002914 10.1002/adfm.202002914.

[ref20] Liu X.; Li M.; Zheng X.; Retulainen E.; Fu S. Dual Light- and pH-Responsive Composite of Polyazo-Derivative Grafted Cellulose Nanocrystals. Materials (Basel) 2018, 11, 1725 10.3390/ma11091725. 30223462 PMC6165044

[ref21] Ceamanos L.; Kahveci Z.; Lopez-Valdeolivas M.; Liu D.; Broer D. J.; Sanchez-Somolinos C. Four-Dimensional Printed Liquid Crystalline Elastomer Actuators with Fast Photoinduced Mechanical Response toward Light-Driven Robotic Functions. ACS Appl. Mater. Interfaces 2020, 12, 44195–44204. 10.1021/acsami.0c13341. 32885661

[ref22] Wagner N.; Theato P. Light-Induced Wettability Changes on Polymer Surfaces. Polymer 2014, 55, 3436–3453. 10.1016/j.polymer.2014.05.033.

[ref23] Kim Y.; Jeong D.; Shinde V. V.; Hu Y.; Kim C.; Jung S. Azobenzene-Grafted Carboxymethyl Cellulose Hydrogels with Photo-Switchable, Reduction-Responsive and Self-Healing Properties for a Controlled Drug Release System. Int. J. Biol. Macromol. 2020, 163, 824–832. 10.1016/j.ijbiomac.2020.07.071. 32653370

[ref24] Poprawa-Smoluch M.; Baggerman J.; Zhang H.; Maas H. P. A.; De Cola L.; Brouwer A. M. Photoisomerization of Disperse Red 1 Studied with Transient Absorption Spectroscopy and Quantum Chemical Calculations. J. Phys. Chem. A 2006, 110, 11926–11937. 10.1021/jp054982b. 17064180

[ref25] Tang R.; Wen J.; Stote R. E.; Sun Y. Cyanuric Chloride-Based Reactive Dyes for Use in the Antimicrobial Treatments of Polymeric Materials. ACS Appl. Mater. Interfaces 2021, 13, 1524–1534. 10.1021/acsami.0c18613. 33378153

[ref26] Prabhaharan M.; Prabakaran A. R.; Srinivasan S.; Gunasekaran S. Experimental and Theoretical Spectroscopic Analysis, HOMO-LUMO, and NBO Studies of Cyanuric Chloride. Spectrochim. Acta - Part A Mol. Biomol. Spectrosc. 2014, 127, 454–462. 10.1016/j.saa.2014.02.040. 24650880

[ref27] Sun Y.; Chen Z.; Braun M. Preparation and Physical and Antimicrobial Properties of a Cellulose-Supported Chloromelamine Derivative. Ind. Eng. Chem. Res. 2005, 44, 7916–7920. 10.1021/ie0504452.

[ref28] Cinar M.; Coruh A.; Karabacak M. FT-IR, UV-Vis, 1H and 13C NMR Spectra and the Equilibrium Structure of Organic Dye Molecule Disperse Red 1 Acrylate: A Combined Experimental and Theoretical Analysis. Spectrochim. Acta - Part A Mol. Biomol. Spectrosc. 2011, 83, 561–569. 10.1016/j.saa.2011.09.003. 21958518

[ref29] Xiao L.; Mai Y.; He F.; Yu L.; Zhang L.; Tang H.; Yang G. Bio-Based Green Composites with High Performance from Poly(Lactic Acid) and Surface-Modified Microcrystalline Cellulose. J. Mater. Chem. 2012, 22, 15732–15739. 10.1039/c2jm32373g.

[ref30] Goulet-Hanssens A.; Corkery T. C.; Priimagi A.; Barrett C. J. Effect of Head Group Size on the Photoswitching Applications of Azobenzene Disperse Red 1 Analogues. J. Mater. Chem. C 2014, 2, 7505–7512. 10.1039/C4TC00996G.

[ref31] French A. D. Idealized Powder Diffraction Patterns for Cellulose Polymorphs. Cellulose 2014, 21, 885–896. 10.1007/s10570-013-0030-4.

[ref32] Gong J.; Li J.; Xu J.; Xiang Z.; Mo L. Research on Cellulose Nanocrystals Produced from Cellulose Sources with Various Polymorphs. RSC Adv. 2017, 7, 33486–33493. 10.1039/C7RA06222B.

[ref33] Banerjee M.; Saraswatula S.; Williams A.; Brettmann B. Effect of Purification Methods on Commercially Available Cellulose Nanocrystal Properties and TEMPO Oxidation. Processes 2020, 8, 698 10.3390/pr8060698.

[ref34] Agarwal U. P.; Ralph S. A.; Baez C.; Reiner R. S.; Verrill S. P. Effect of Sample Moisture Content on XRD-Estimated Cellulose Crystallinity Index and Crystallite Size. Cellulose 2017, 24, 1971–1984. 10.1007/s10570-017-1259-0.

[ref35] Park S.; Baker J. O.; Himmel M. E.; Parilla P. A.; Johnson D. K. Cellulose Crystallinity Index: Measurement Techniques and Their Impact on Interpreting Cellulase Performance. Biotechnol. Biofuels 2010, 3, 10 10.1186/1754-6834-3-10. 20497524 PMC2890632

[ref36] Fernandes Diniz J. M. B.; Gil M. H.; Castro J. A. A. M. Hornification - Its Origin and Interpretation in Wood Pulps. Wood Sci. Technol. 2004, 37, 489–494. 10.1007/s00226-003-0216-2.

[ref37] Sultan S.; Siqueira G.; Zimmermann T.; Mathew A. P. 3D Printing of Nano-Cellulosic Biomaterials for Medical Applications. Curr. Opin. Biomed. Eng. 2017, 2, 29–34. 10.1016/j.cobme.2017.06.002.

[ref38] Pinto F.; Meo M. Design and Manufacturing of a Novel Shear Thickening Fluid Composite (STFC) with Enhanced out-of-Plane Properties and Damage Suppression. Appl. Compos. Mater. 2017, 24, 643–660. 10.1007/s10443-016-9532-1.

[ref39] Krieger I. M.; Dougherty T. J. A Mechanism for Non-Newtonian Flow in Suspensions of Rigid Spheres. Trans. Soc. Rheol. 1959, 3, 137–152. 10.1122/1.548848.

[ref40] Le E. A.; Wang W. C.; Liu C. S.; Wang C. W. Effect of Fiber Alignment on Optical Properties of Cellulose Nanocrystal Films. Proc. Int. Symp. Optomechatronic Technol. 2014, 305–309. 10.1109/ISOT.2014.80.

[ref41] Cranston E. D.; Gray D. G. Birefringence in Spin-Coated Films Containing Cellulose Nanocrystals. Physicochem. Eng. Asp. 2008, 325, 44–51. 10.1016/j.colsurfa.2008.04.042.

[ref42] Eremeeva E.; Sergeeva E.; Neterebskaia V.; Morozova S.; Kolchanov D.; Morozov M.; Chernyshov I.; Milichko V.; Vinogradov A. Printing of Colorful Cellulose Nanocrystalline Patterns Visible in Linearly Polarized Light. ACS Appl. Mater. Interfaces 2020, 12, 45145–45154. 10.1021/acsami.0c11846. 32816443

[ref43] Müller L. A. E.; Demongeot A.; Vaucher J.; Leterrier Y.; Avaro J.; Liebi M.; Neels A.; Burgert I.; Zimmermann T.; Nyström G.; Siqueira G. Photoresponsive Movement in 3D Printed Cellulose Nanocomposites. ACS Appl. Mater. Interfaces 2022, 14, 16703 10.1021/acsami.2c02154. 35377597

[ref44] Vapaavuori J.; Laventure A.; Bazuin C. G.; Lebel O.; Pellerin C. Submolecular Plasticization Induced by Photons in Azobenzene Materials. J. Am. Chem. Soc. 2015, 137, 13510–13517. 10.1021/jacs.5b06611. 26439981

[ref45] Burgert I.; Frühmann K.; Keckes J.; Fratzl P.; Stanzl-Tschegg S. E. Microtensile Testing of Wood Fibers Combined with Video Extensometry for Efficient Strain Detection. Holzforschung 2003, 57, 661–664. 10.1515/HF.2003.099.

[ref46] Huang W. M.; Yang B.; Fu Y. Q. Polyurethane Shape Memory Polymers; CRC Press: Boca Raton, London, Newyork, 2011 10.1201/b11209.

[ref47] Chien Y. C.; Chuang W. T.; Jeng U. S.; Hsu S. H. Preparation, Characterization, and Mechanism for Biodegradable and Biocompatible Polyurethane Shape Memory Elastomers. ACS Appl. Mater. Interfaces 2017, 9, 5419–5429. 10.1021/acsami.6b11993. 28165708

[ref48] Xie F.; Huang L.; Leng J.; Liu Y. Thermoset Shape Memory Polymers and Their Composites. J. Intell. Mater. Syst. Struct. 2016, 27, 2433–2455. 10.1177/1045389X16634211.

[ref49] Ratna D.; Karger-Kocsis J. Recent Advances in Shape Memory Polymers and Composites: A Review. J. Mater. Sci. 2008, 43, 254–269. 10.1007/s10853-007-2176-7.

[ref50] Chu H.; Yang W.; Sun L.; Cai S.; Yang R.; Liang W.; Yu H.; Liu L. 4D Printing: A Review on Recent Progresses. Micromachines 2020, 11, 796 10.3390/mi11090796. 32842588 PMC7570144

[ref51] Gibson L. J.; Ashby M. F. Cellular Solids: Structure & Properties; Pergamon Press: Oxford, 1989.

[ref52] Correa D. M.; Seepersad C. C.; Haberman M. R. Mechanical Design of Negative Stiffness Honeycomb Materials. Integr. Mater. Manuf. Innov. 2015, 4, 165–175. 10.1186/s40192-015-0038-8.

[ref53] Debeau D. A.; Seepersad C. C.; Haberman M. R. Impact Behavior of Negative Stiffness Honeycomb Materials. J. Mater. Res. 2018, 33, 290–299. 10.1557/jmr.2018.7.

[ref54] Chen S.; Tan X.; Hu J.; Zhu S.; Wang B.; Wang L.; Jin Y.; Wu L. A Novel Gradient Negative Stiffness Honeycomb for Recoverable Energy Absorption. Compos. Part B Eng. 2021, 215, 108745 10.1016/j.compositesb.2021.108745.

[ref55] Correa D. M.; Klatt T.; Cortes S.; Haberman M.; Kovar D.; Seepersad C. Negative Stiffness Honeycombs for Recoverable Shock Isolation. Rapid Prototyp. J. 2015, 21, 193–200. 10.1108/RPJ-12-2014-0182.

[ref56] Arcari M.; Zuccarella E.; Axelrod R.; Adamcik J.; Sánchez-Ferrer A.; Mezzenga R.; Nyström G. Nanostructural Properties and Twist Periodicity of Cellulose Nanofibrils with Variable Charge Density. Biomacromolecules 2019, 20, 1288–1296. 10.1021/acs.biomac.8b01706. 30673281

[ref57] Beamson G.; Briggs D. High Resolution XPS of Organic Polymers: The Scienta ESCA300 Database. Wiley: New York, 1992.

