# Supplementary material for: Functionalized Cellulose Nanocrystals as Active Reinforcements for Light-Actuated 3D-Printed Structures
Source: ACS Nano. 2022 Oct 18;16(11):18210–22. doi: 10.1021/acsnano.2c05628 (PMC9706808; doi:10.1021/acsnano.2c05628)
Supplement: Supplementary file 1 — nn2c05628_si_001.pdf [file nn2c05628_si_001.pdf]

Supporting Information

Functionalized Cellulose Nanocrystals as Active Reinforcements for Light Actuated 3D Printed Structures

Luca A. E. Müller,[1,2+] Anita Zingg,[1,2+] Andrea Arcifa,[3] Tanja Zimmermann,[1] Gustav Nyström,[1,4] Ingo Burgert,[1,2] Gilberto Siqueira[1]**

[+]*Shared first co-authorship*

1 Cellulose and Wood Materials Laboratory, Empa, Swiss Federal Laboratories for Materials Science and Technology, 8600 Dübendorf, Switzerland.

2 Wood Materials Science, Institute for Building Materials, ETH-Zürich, 8093 Zürich, Switzerland.

3 Surface Science & Coating Technologies, Empa, Empa, Swiss Federal Laboratories for Materials Science and Technology, 8600 Dübendorf, Switzerland.

4 Department of Health Sciences and Technology, ETH Zürich, 8092 Zürich, Switzerland.

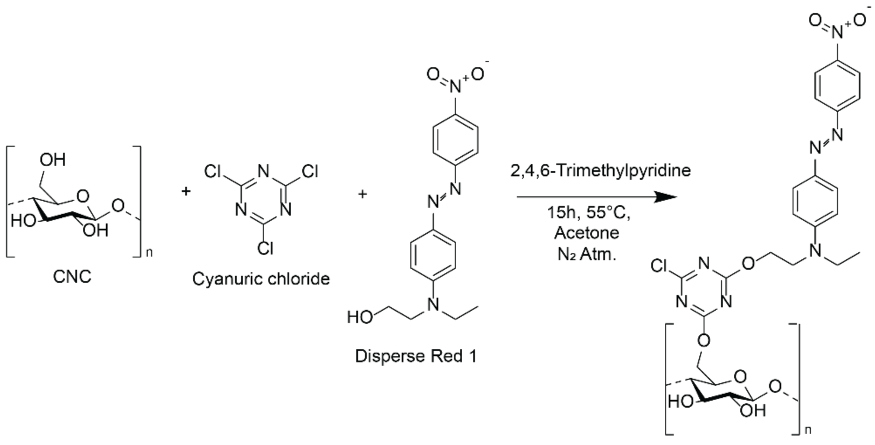

Figure S1. *Schematic representation of the chemical modification of CNC. The grafting is represented here on C6, accounting for the higher probability that the reaction occurs in this position due to the reduced steric hinderance of this group. However, the reaction could also occur on the other OH groups of the AGU (Anhydroglucose Unit).*

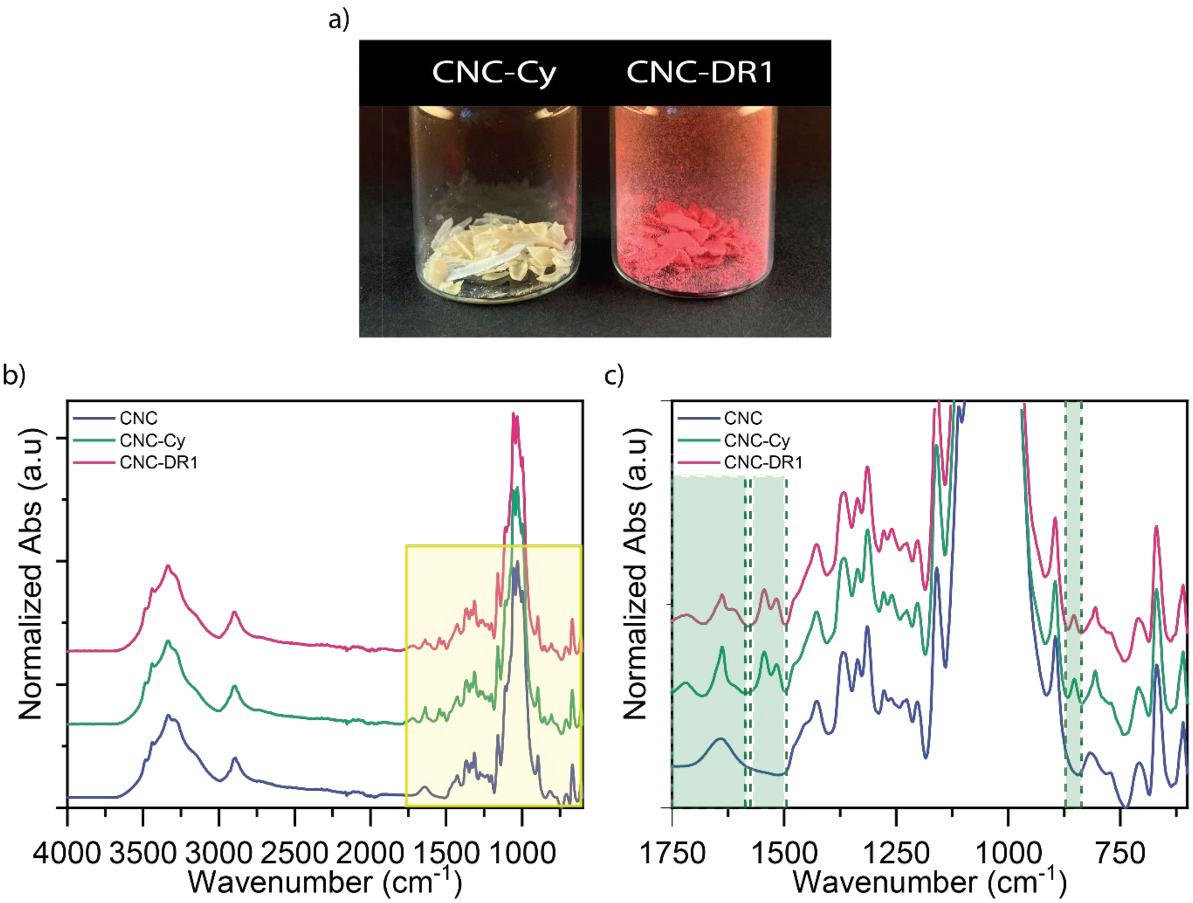

Figure S2. *ATR-FTIR Spectra of the CNC before, intermediate and after modification. (**a**)Photograph of the two reaction products CNC-Cy (modified only with cyanuric chloride) and CNC-DR1. (**b**) Full spectra including CNC, CNC-Cy, and modified CNC-DR1. (**c**) Enlargement of the yellow region of the spectra in (b) between 1750 and 600 cm^{-1}, green boxes highlight the regions of interest in the spectra.*

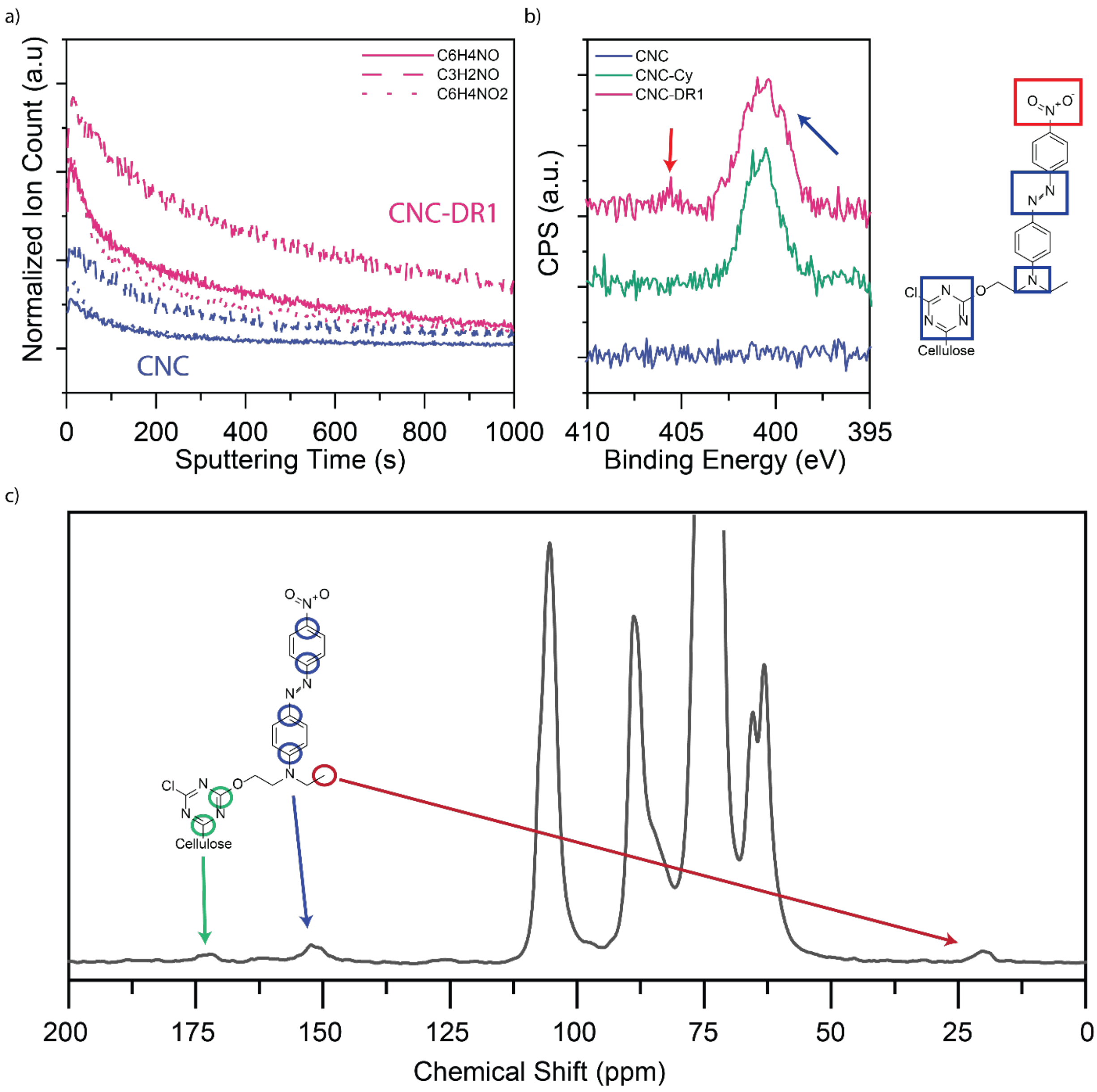

Figure S3. *Chemical characterization of CNC-DR1. (**a**) DR1ToF-SIMS negative spectra of CNC-DR1 and CNC for the NO_2 group of DR1 Azobenzene. (**b**) XPS spectra of N1s signal for CNC, CNC-Cy, CNC-DR1 (left) and the grafted DR1 compound (right) the colored arrows indicates which peak corresponds to which N atoms in the compound. (**c**) Solid state C^{13} NMR spectra of CNC-DR1.*

Note on XPS: The DR1 is attached to the cellulose, since ref CNC samples do not show presence of N as contamination. Indeed, nitrogen seems to be present with 1% at. However, the N-O group tends to degrade under X-ray, becoming a N-H group. Longer acquisition times would be required

to have a full quantification, but this would lead to destruction of the functional group and decreasing of the NO_2 peak at 405-406 eV.

Estimation of Substitution fraction of CNC accessible hydroxyls from Solid State NMR: The number of OH reacting with DR1 over the number of accessible OH was estimated from the NMR results in Figure S3 by applying the following equation:[1,2]

$$\omega = \frac{\frac{\int C_{Dr1}}{4}}{0.45 * 3 * \int C1_{CNC}} = 0.7\%$$

Where $\int C_{Dr1}$ corresponds to the area of the four aromatic carbons of DR1 at 152 ppm, while $\int C1_{CNC}$ corresponds to the area of the C1 of the anhydroglucose unite at 105 ppm. Each glucose unit bears 3 hydroxyl and for CNC with a transversal size of around 5 nm, only around 45 % (estimated from Sugiyama et al.[3]) of the total amount of hydroxyl groups is accessible at the surface of the particles.

Table S 1 *Elemental analysis results for CNC and CNC-DR1 obtained with a LECO TruSpec Micro. Samples were dried 24 hours at 60 °C from acetone suspension prior measurement.*

Sample	%C	%H	%Cl	%N	DS	Substitution fraction of CNC accessible hydroxyls
CNC	39.45	5.75	0.61	<0.20	-	-
CNC-DR1	41.99	5.73	2.05	1.80	0.03	2.39 %

Degree of substitution from elemental analysis: The DS per anhydroglucose unit was estimated directly from %N of CNC-DR1 assuming the grafted molecules to be composed of a DR1 and a Cyanuric Chloride unit as illustrated in Figure S1 and that the reference CNC do not contain nitrogen since the measured value is below the detection limit of the instrument. The following equation was applied:[4]

$$DS = \frac{M_n * n_{AGU} - \%N * M_{AGU}}{\%N * M_{com} - M_N * n_N}$$

Where M_N is the nitrogen molecular weight, n_{AGU} the number of nitrogen atoms in an anhydroglucose unit (AGU), M_{AGU} the molecular weight of the AGU, M_{com} is the molecular weight of the grafted compound and n_N is the number of nitrogen atoms in the grafted compound. The substituted fraction of OH groups among those accessible at the surface was obtained by dividing the DS by a factor of 3 (three hydroxyls per AGU) and a factor of 0.45 (fraction of surface hydroxyls).[5]

Note on elemental analysis: The degree of substitution here calculated is likely an over estimation arising from the assumption that one DR1 and one cyanuric chloride are linked together (according to the presence of Cl in the final product and the C-Cl bending band observed from FTIR). However, being a one pot reaction, there is the possibility that more cyanuric chlorides are attached to CNC than DR1. The calculated value remains an estimation.

(1) Xiao, L.; Mai, Y.; He, F.; Yu, L.; Zhang, L.; Tang, H.; Yang, G. Bio-Based Green Composites with High Performance from Poly(Lactic Acid) and Surface-Modified Microcrystalline Cellulose. *J. Mater. Chem.* **2012**, *22* (31), 15732–15739. https://doi.org/10.1039/c2jm32373g.

(2) Gårdebjer, S.; Bergstrand, A.; Idström, A.; Börstell, C.; Naana, S.; Nordstierna, L.; Larsson, A. Solid-State NMR to Quantify Surface Coverage and Chain Length of Lactic Acid Modified Cellulose Nanocrystals, Used as Fillers in Biodegradable Composites. *Compos. Sci. Technol.* **2015**, *107*, 1–9. https://doi.org/10.1016/j.compscitech.2014.11.014.

(3) Sugiyama, J.; Vuong, R.; Chanzy, H. Electron Diffraction Study on the Two Crystalline Phases Occurring in Native Cellulose from an Algal Cell Wall. *Macromolecules* **1991**, *24* (14), 4168–4175. https://doi.org/10.1021/ma00014a033.

(4) Vaca-Garcia, C.; Borredon, M. E.; Gaseta, & A. *Determination of the Degree of Substitution (DS) of Mixed Cellulose Esters by Elemental Analysis*; 2001; Vol. 8.

(5) Siqueira, G.; Bras, J.; Dufresne, A. New Process of Chemical Grafting of Cellulose Nanoparticles with a Long Chain Isocyanate. *Langmuir* **2010**, *26* (1), 402–411. https://doi.org/10.1021/la9028595.

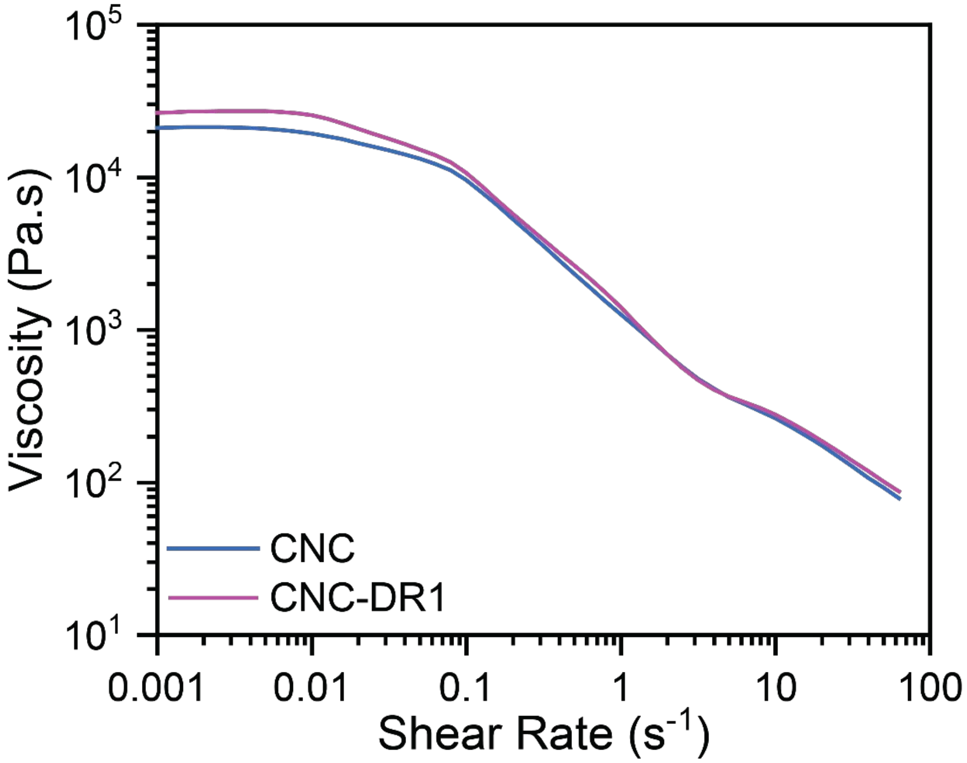

Figure S4, *Shear thinning behavior of 15 wt% CNC or CNC-DR1 in PUA-HEMA inks. Rheology measurements obtained with a rotational experiment.*

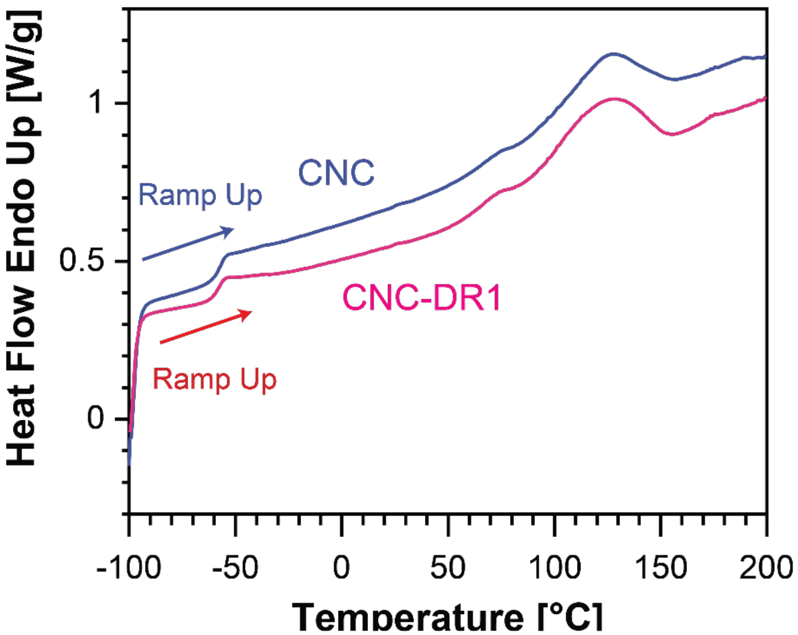

Figure S5. *DSC analysis of composites with 15 wt% of particles in the PUA-HEMA matrix for both CNC and CNC-DR1. First heating cycle obtained at 20 °C min*$^{-1}$.

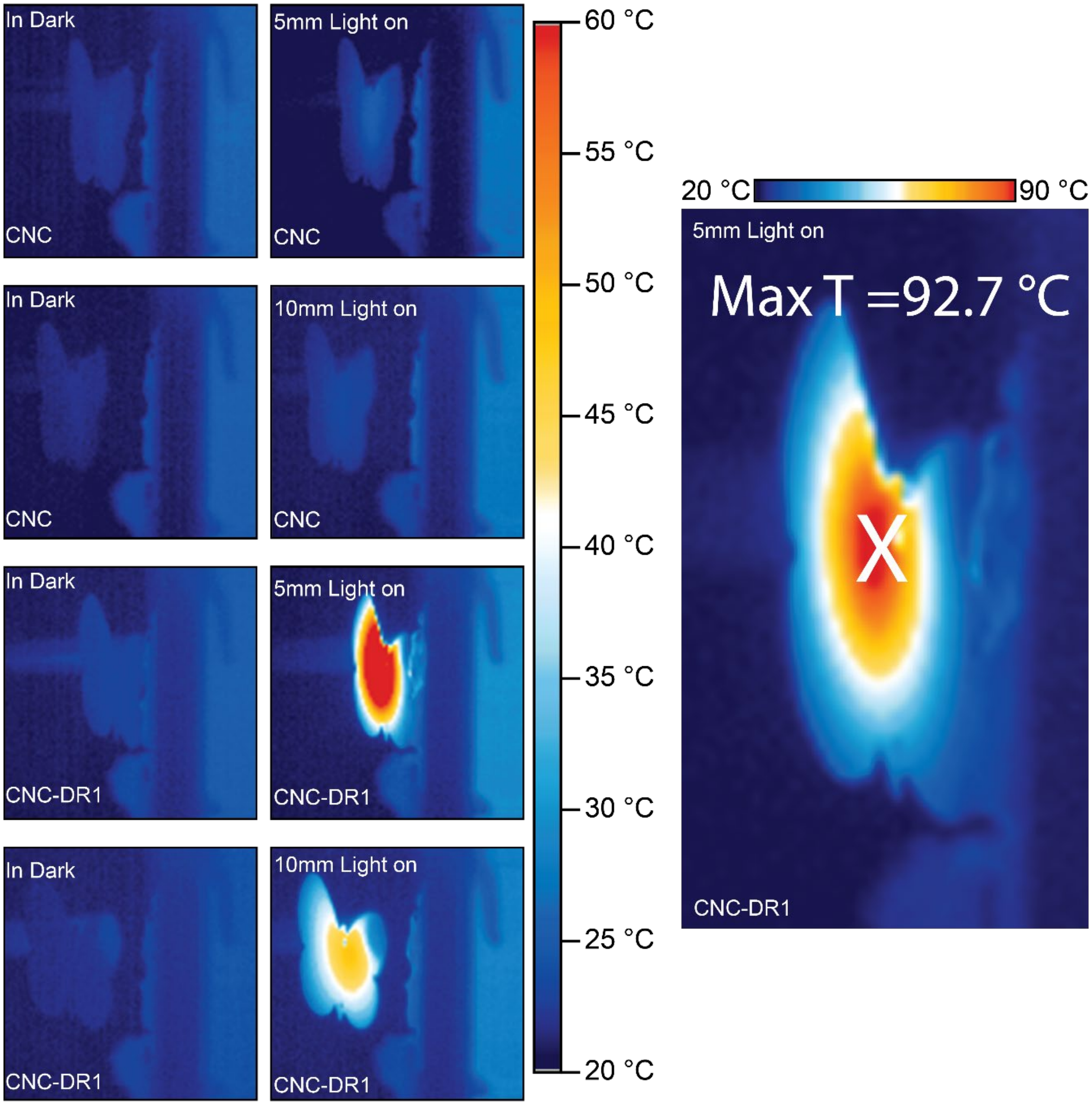

Figure S6. *Thermographs of the printed composite materials in dark or under illumination. The enlargement (right image) is the CNC-DR1 composite illuminated at 5 mm distance with the adjusted scale.*

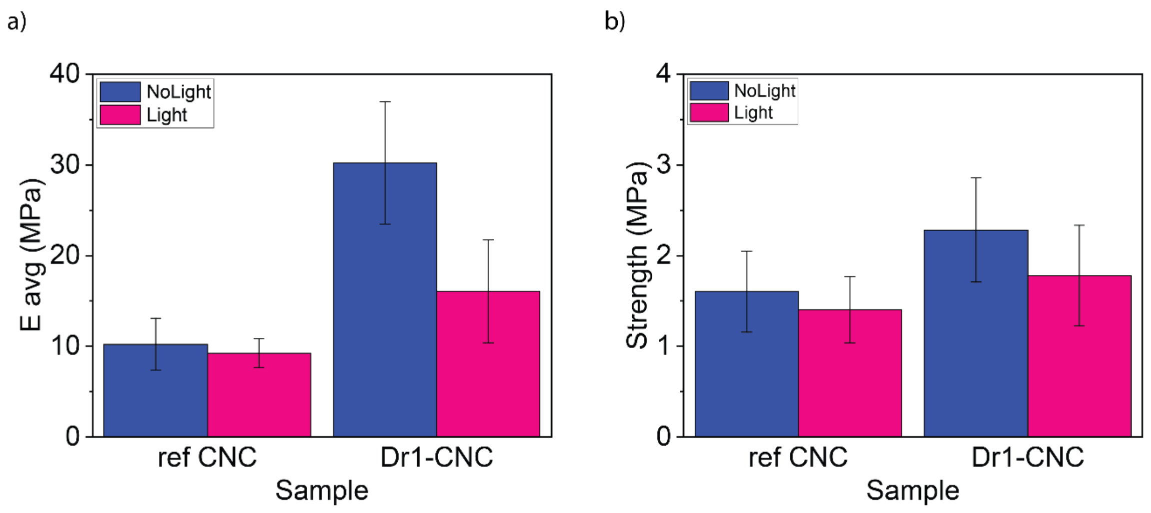

Figure S7. *Mechanical properties of the tested composites. (**a**) Young moduli and (**b**) strength extracted from the microtensile tests.*

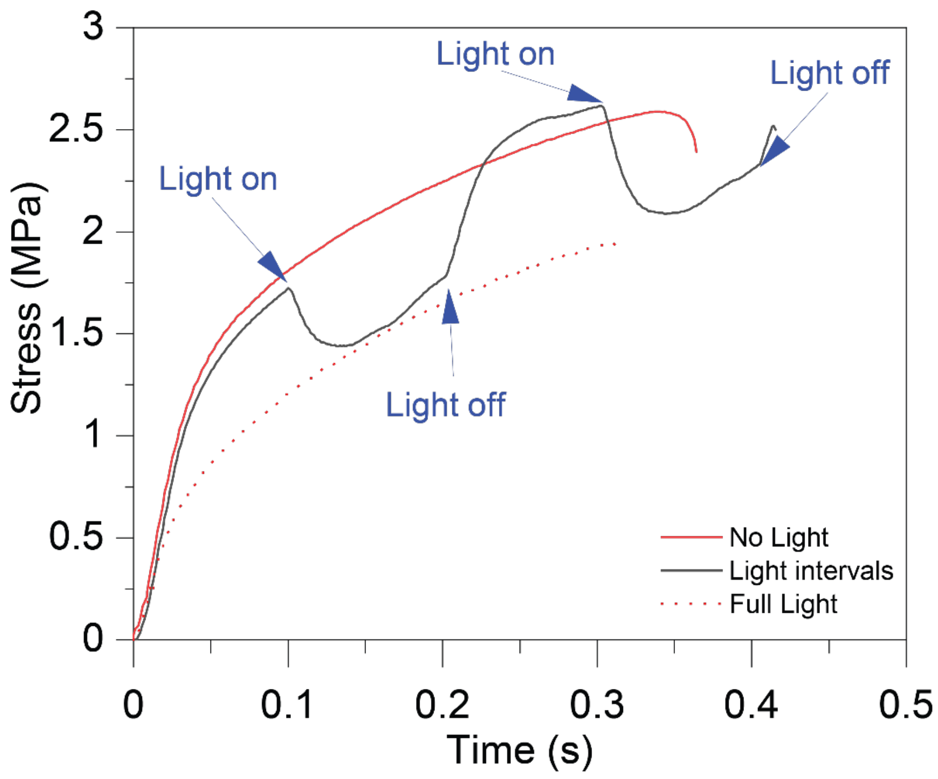

Figure S8. *Microtensile tests of CNC-DR1 composites in dark, under illumination and under selective light intervals.*

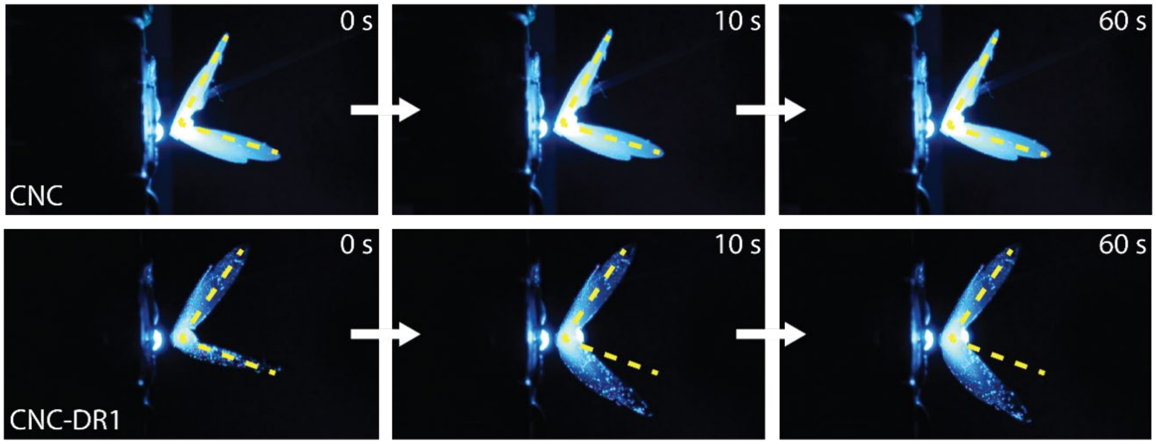

Figure S9. *Comparison of the shape memory effect triggered by illumination for CNC **(a)** and unmodified CNC-DR1 **(b)** printed composites.*

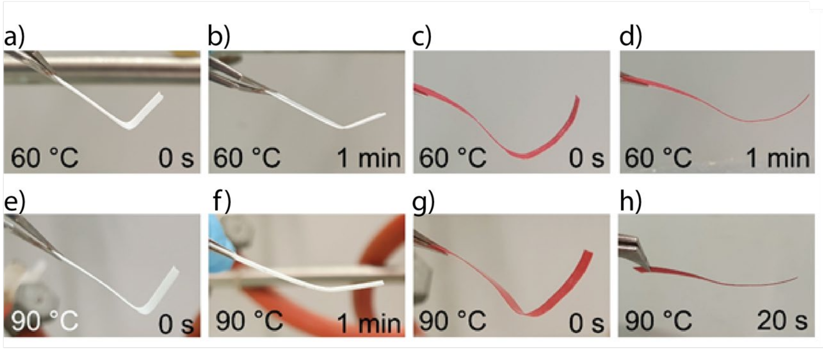

Figure S10. *Thermally activated shape memory effect of the printed composites for CNC **(a,b,e,f)** and CNC-DR1 **(c,d,g,h)**.*

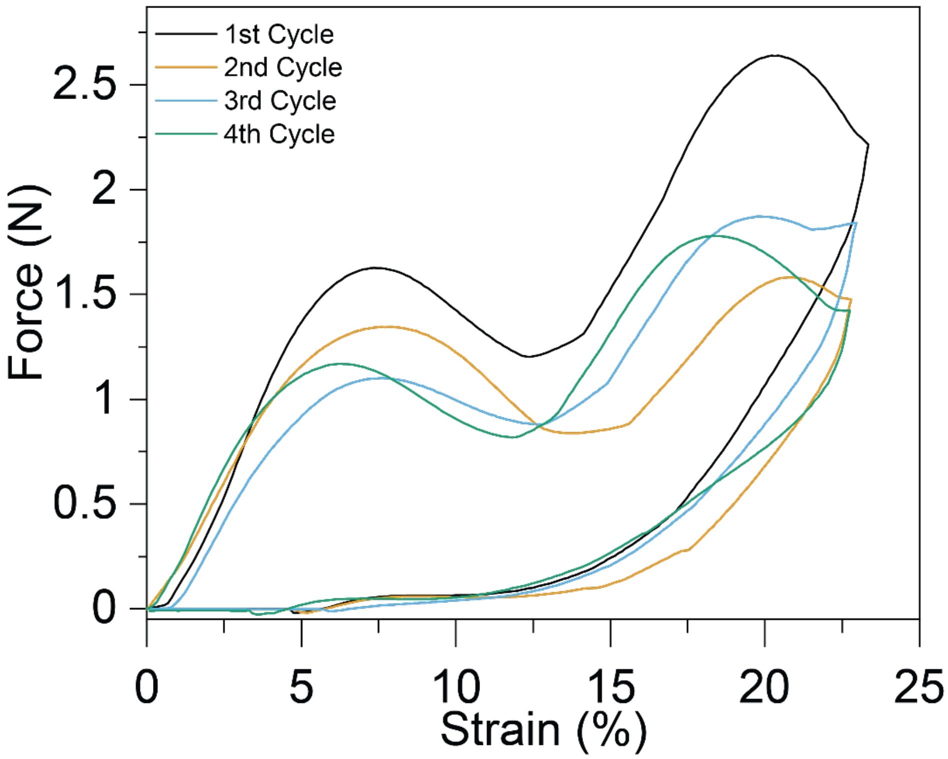

Figure S11. *Compression of a CNC-DR1 composite printed as negative stiffness honeycomb. The sample was illuminated for 5 minutes after each cycle in order to recover its initial shape.*
